# Harnessing Machine Learning for Accelerated Drug Discovery: Opportunities and Unmet Challenges

**DOI:** 10.3390/ph19060810

**Published:** 2026-05-22

**Authors:** Mohamed El-Tanani, Syed Arman Rabbani, Adil Farooq Wali, Frezah Muhana, Yahia El-Tanani, Rakesh Kumar

**Affiliations:** 1 RAK College of Pharmacy, Ras Al Khaimah Medical and Health Sciences University, Ras Al Khaimah 11172, United Arab Emirates; arman@rakmhsu.ac.ae (S.A.R.);; 2 Princess Sarvath Community College, Amman 11196, Jordan; 3 Royal Cornwall Hospital Trust, NHS, Truro TR1 3LJ, UK; 4 Amity Institute of Pharmacy, Amity University, Panchgaon, Gurgaon 122412, India; 5 Department of Pharmacy, Jagannath University, Bahadurgarh 124507, India

**Keywords:** drug discovery, machine learning, artificial intelligence, generative models, computational chemistry, lead optimization, multimodal foundation models, regulatory frameworks

## Abstract

The process of drug discovery is one of the most expensive, time-consuming, and high-risk endeavors in modern science. Translating initial scientific insights into safe and effective therapies, supported by genomics, structural biology, and computational chemistry, typically requires more than a decade and substantial financial investment. Machine learning (ML) has emerged as a powerful tool for improving efficiency across the drug discovery pipeline. By enabling the analysis of large and complex datasets, ML supports target identification, lead discovery, optimization, and prediction of preclinical and clinical outcomes. Its integration with experimental validation and automation is illustrated by recent advances such as protein structure prediction, AI-driven antifibrotic compound discovery, and antibiotic identification. Despite these advances, significant challenges remain. Model generalizability is limited by data scarcity, heterogeneity, and hidden biases. In addition, the translation of in silico predictions into clinically validated outcomes remains a major bottleneck, and regulatory acceptance is constrained by limited model interpretability. Ethical considerations, including data privacy, equitable representation, and the potential misuse of generative models, further complicate adoption. This review examines the applications of ML across the drug discovery pipeline, with a focus on translational and regulatory considerations. It also discusses emerging directions, including hybrid physics–AI approaches, multimodal foundation models, federated learning, and explainable AI. The effective integration of ML will depend on rigorous validation, interdisciplinary collaboration, responsible data governance, and alignment with regulatory frameworks.

## 1. Introduction

The discovery of new medicines is fundamental to modern medical practice, yet it remains an extremely challenging, resource-intensive, and high-risk scientific endeavor [1,2]. This process translates basic biological insights into therapeutic interventions. Despite advances in genomics, proteomics, screening technologies, and computational chemistry, drug development continues to be slow and costly. On average, it takes 10–15 years for a drug to progress from initial discovery to regulatory approval, with costs exceeding $2.5 billion and continuing to rise [3,4]. Moreover, success rates remain low, with only about 10% of clinical analogs achieving approval, and most fail in late-stage trials due to safety or efficacy concerns [5,6].

To address these challenges, the pharmaceutical industry and research community are increasingly turning to machine learning (ML) as a transformative approach [7]. ML enables the identification of complex, non-linear patterns within large, multidimensional datasets, supporting predictive modeling, hypothesis generation, and compound design [8]. This capability is particularly valuable in drug discovery, where diverse data types, including chemical structures, biological assays, genomic data, clinical records, and imaging, must be integrated and analyzed [9].

Recent high-profile successes have further accelerated interest in ML-driven drug discovery. Notable examples include AlphaFold for protein structure prediction, AI-generated drug candidates from Insilico Medicine, and large-scale virtual screening approaches developed by Atomwise [10,11]. These advances highlight the potential of ML to accelerate discovery timelines and expand our understanding of chemical and biological systems [12].

Despite this promise, most ML applications in drug discovery remain at the proof-of-concept or early validation stage [13,14]. Translating computational predictions into clinically viable drugs remains difficult. Key challenges include limited and noisy datasets, methodological limitations, and issues related to reproducibility and generalizability [9,14]. In addition, regulatory agencies such as the U.S. Food and Drug Administration and the European Medicines Agency require transparency, interpretability, and rigorous validation before adopting AI-based approaches in drug development [15].

The performance of ML models is highly dependent on data quality. Many of the most valuable chemical and biological data remain proprietary, limiting accessibility [9,16]. Public databases such as ChEMBL, PubChem, and BindingDB provide important resources but are often affected by inconsistencies, incomplete annotations, and variability in experimental conditions [17,18,19]. These limitations reduce reliability and increase the risk of overfitting, ultimately affecting predictive accuracy [20].

Beyond technical challenges, organizational and cultural barriers also hinder the adoption of ML in drug discovery. Traditional workflows rely heavily on expert-driven decision-making in medicinal chemistry and pharmacology. Integrating ML into these workflows requires not only technological adaptation but also changes in mindset and training. Concerns regarding model transparency, reproducibility, and regulatory acceptance continue to slow adoption [17,18,19]. Building trust will require improved interpretability, interdisciplinary collaboration, and demonstration of consistent real-world success.

Despite these limitations, the integration of ML with emerging technologies offers a promising path forward. Advances in automation, robotics, and synthetic biology are enabling closed-loop discovery systems in which ML-generated hypotheses can be rapidly tested and refined [20,21]. Similarly, the integration of multi-omics data and clinical information supports improved patient stratification and personalized treatment strategies [22]. Developments in foundation models, federated learning, and explainable AI are also helping to address existing technical and ethical challenges.

Several recent publications have discussed computational, AI-assisted, and data-driven approaches in modern drug discovery, including applications involving virtual screening, molecular property prediction, generative chemistry, explainable AI, and computer-aided drug design [23,24]. These studies collectively demonstrate the growing impact of ML and AI across multiple stages of pharmaceutical research, ranging from target identification and hit discovery to optimization and toxicity prediction. Earlier reviews have provided valuable overviews of deep learning architectures, cheminformatics workflows, predictive modeling strategies, and emerging AI platforms designed to accelerate therapeutic development [25,26]. Other reports have focused on explainable AI, molecular representation methods, and the integration of large biomedical datasets for computational drug discovery. Together, these contributions establish the importance of AI-driven methodologies as increasingly influential tools within contemporary pharmaceutical science [26,27].

Despite these advances, several important translational and medicinal chemistry challenges remain comparatively underexplored in the existing review literature. In particular, many current discussions emphasize algorithm performance and benchmark accuracy while giving less attention to practical decision-making constraints encountered during real-world lead optimization and candidate selection [27,28]. The present work therefore places stronger emphasis on selectivity optimization, SAR continuity, activity cliffs, scaffold hopping, synthesizability, route feasibility, developability constraints, and uncertainty-aware prediction within medicinal chemistry workflows [29,30]. In addition, hybrid physics–AI strategies, including ML-enhanced molecular dynamics, AI-corrected docking, QM/MM workflows, and foundation-model-based approaches, are critically evaluated alongside issues related to reproducibility, prospective validation, regulatory expectations, and translational reliability [30]. Rather than presenting ML solely as a technological acceleration tool, the discussion focuses on how computational predictions can meaningfully support experimentally grounded drug discovery decisions under practical pharmaceutical development constraints [31,32]. Collectively, this perspective aims to provide medicinal chemists, computational scientists, pharmacologists, translational researchers, and regulatory stakeholders with a balanced and practice-oriented assessment of the opportunities, current limitations, and unmet challenges associated with ML-driven drug discovery.

## 2. Overview of Machine Learning in Drug Discovery

From the discovery of penicillin to the design of kinase inhibitors, the development of new medications has followed a continuous path of advancement, with new technologies and techniques driving each stage of development [20,21,22]. Quantitative structure–activity relationship (QSAR) models and molecular docking techniques gave rise to the first computer tools for algorithmic prediction, but due to their linear modeling approaches and constrained data access, their functionality remained limited [33,34]. Although the operational range of these systems remained within recognized patterns of chemical knowledge, they showed success in predicting molecular interactions.

Data-driven discovery underwent an enormous transformation with the advent of machine learning (ML). The identification of intricate, non-linear correlations contained in large datasets, which was challenging using conventional techniques, was made possible using machine learning techniques [35,36]. In order to anticipate parameters like solubility, binding affinity, and toxicity, early applications mostly used supervised learning and labeled datasets. Despite their effectiveness, these models needed reliable, high-quality annotated data [37,38,39,40].

Graph-based deep learning approaches have further improved molecular representation, particularly through graph neural networks (GNNs), which operate directly on molecular graphs where atoms and bonds are encoded as nodes and edges. Unlike traditional descriptor-based methods that rely on predefined features, GNNs learn task-specific representations by propagating information across molecular structures, capturing both local chemical environments and long-range dependencies critical for structure–activity relationships [41]. In parallel, generative models such as variational autoencoders (VAEs) enable molecular optimization by embedding compounds into a continuous latent space, where structurally related molecules cluster together. This allows systematic navigation toward regions associated with improved properties (e.g., potency or ADMET profiles) using optimization strategies, such as gradient-based search or reinforcement learning, before decoding back into candidate structures [39,40,41,42]. Diffusion-based models extend this paradigm by iteratively refining molecular representations through denoising processes, improving diversity and stability of generated compounds [40].

Unsupervised learning methods further expanded analytical capabilities by identifying hidden patterns in large chemical datasets through clustering and dimensionality reduction techniques [36,37]. These methods succeeded in exploring chemical space outside of human intuition. Reinforcement learning (RL) introduced additional flexibility by framing molecular design as an optimization problem. In order to improve efficacy and safety profiles, agents iteratively alter molecular structures based on drive signals [38]. The development of generative models marked a major breakthrough in de novo molecular design, enabling the creation of novel compounds beyond the known chemical space through approaches such as VAEs, GANs, and diffusion models [40].

**Figure 1:**
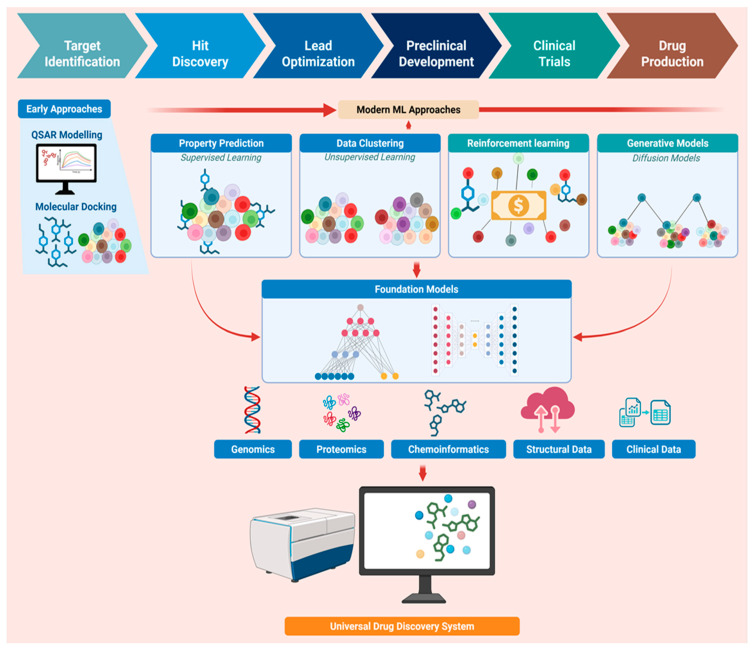
Overview of machine learning in drug discovery. The conceptual schematic demonstrates how machine learning (ML) applications in drug discovery have evolved to assist drug development from discovery through production. The figure demonstrates the development of computational methods, which started with QSAR modeling and molecular docking before moving to present-day AI-based systems. The research progress includes supervised learning for property prediction, unsupervised learning for data clustering, reinforcement learning for molecular design, and generative models (VAEs, GANs, diffusion models) for de novo compound creation. The diagram demonstrates how foundation models process various biomedical data types, which enable the development of universal drug discovery systems, defined here as integrated, multi-task AI frameworks capable of performing target identification, molecular design, and predictive evaluation within a unified pipeline (Created in BioRender. El-Tanani, M. (2026) https://BioRender.com/ui9bk2w accessed on 28 April 2026).

Recent drug discovery research integrates computational intelligence with multiple frameworks. ML has progressed from prediction tools to more specific domains for a new class of foundation models that are trained on diverse datasets, encompassing molecular, protein, and biological information [41,42]. These models offer potential to perform multiple tasks, including drug candidate generation, evaluation, and optimization [43]. Despite progress in AI for drug discovery, issues regarding interpretation, experimental validation, and data transparency continue to limit its widespread adoption [43,44] (Figure 1).

## 3. Opportunities for Acceleration

Machine learning enables acceleration across multiple stages of drug discovery by improving data integration, predictive modeling, and hypothesis generation [44,45].

### 3.1. Target Identification and Validation

Identifying appropriate biological targets is a critical first step in drug discovery, as therapeutic success depends on precise modulation of disease-associated biomolecules. Traditionally, target identification relied on experimental methods and serendipitous discoveries, requiring extensive laboratory effort and biological insight [46,47]. Machine learning technology has revolutionized drug discovery by integrating massive datasets, from genomic to clinical domains, identifying new target associations at high speed and large scale [48].

Graph neural networks (GNNs) and knowledge-graph-based methods have shown effectiveness in analyzing disease networks and identifying potential therapeutic targets that may be overlooked by conventional approaches [49]. In parallel, natural language processing (NLP) models enable extraction of relevant information from scientific literature and electronic health records. They support the identification of gene–disease relationships and therapeutic possibilities [50]. Despite these advances, experimental validation remains essential for confirming disease relevance, safety, and efficacy before further development [51,52].

### 3.2. Hit Identification

Hit identification focuses on the selection of target molecules show strong and specific binding to the chosen biological target. The traditional method of high-throughput screening (HTS) used to test millions of compounds, but this process was both expensive and time-consuming. The current use of machine learning (ML) technology enables virtual screening, which functions as a virtual screening method that replaces physical assays to evaluate large chemical libraries [53,54]. The combination of generative models with deep neural networks allows scientists to create new chemical compounds for particular target properties while simultaneously predicting their binding capabilities with high precision [53,54,55]. The AI-based screening platforms developed by Atomwise and BenevolentAI have shown success in executing large-scale screening for promising candidate compounds to treat oncological and infectious diseases [55]. The most prominent challenge with ML-generated compounds is their lack of synthetic accessibility, requiring additional cheminformatic evaluation to validate their potential before practical manufacturing [56].

### 3.3. Lead Optimization

ML approaches support lead optimization by enabling data-driven refinement of molecular candidates to balance efficacy, selectivity, and safety. The optimization of drugs with improved selectivity and ADMET profiles through ML frameworks allows scientists to shift from the traditional method of trial-and-error to data-driven molecular optimization [56]. Multi-objective optimization techniques allow simultaneous consideration of multiple parameters, which help to improve therapeutic performance while minimizing toxicity [57]. These models integrate computational predictions with experimental feedback to prioritize compounds for synthesis and testing, thereby reducing experimental cycles and improving efficiency [58].

#### 3.3.1. Medicinal Chemistry Decision Context

A practical medicinal chemistry campaign is not organized around a single activity prediction; instead, it is organized around compound series, assay cycles, structure-based hypotheses, synthetic constraints, and candidate-selection decisions [59]. Therefore, the value of ML in lead optimization should be judged by whether it helps a project team decide which compounds to make next under limited time, budget, and biological information. In real campaigns, a useful model must rank close analogs within a series, compare chemically distinct series, detect when a proposed analog falls outside the model applicability domain, and provide interpretable support for balancing potency, selectivity, ADMET behavior, and developability. This positions ML as a decision-support layer for medicinal chemists rather than as a fully autonomous replacement for expert design [60,61,62].

#### 3.3.2. Selectivity Optimization and Counter-Screening

Selectivity optimization is one of the clearest areas where ML can contribute to medicinal chemistry practice. A compound rarely fails because it is insufficiently active against a single target alone; rather, it may fail because its activity is not selective over homologous proteins, anti-targets, ion channels, cytochrome P450 enzymes, transporter liabilities, or phenotypic liabilities detected in secondary assays [62]. ML can support this problem through multi-task learning across target families, chemogenomic models that learn ligand–target relationships, off-target panel prediction, and active-learning workflows that recommend which counter-screens should be run next. In practice, selectivity should be modeled as a margin between desired and undesired activities, such as a difference in pIC50, pKi, or functional response, rather than as an isolated potency endpoint. This is especially important for kinase inhibitors, GPCR ligands, ion-channel modulators, and antimicrobial discovery, where small structural changes may substantially alter selectivity profiles [60,61,62,63,64].

The limitations are equally important. Off-target datasets are usually sparse, imbalanced, and collected under heterogeneous assay formats. Inactive labels may represent true inactivity, weak activity outside the tested concentration range, or missing experimental follow-up. Consequently, selectivity models should include assay metadata when available, treat censored values cautiously, and report prediction uncertainty. Compounds predicted to have high potency but uncertain selectivity should be prioritized for focused counter-screening rather than advanced directly. This uncertainty-aware use of ML aligns with medicinal chemistry practice because it converts predictions into testable decisions instead of treating them as definitive evidence [42,43,44].

#### 3.3.3. SAR Continuity, Activity Cliffs, and Transferability

SAR continuity is central to medicinal chemistry. Chemists often optimize congeneric analogs because small structural modifications are expected to produce interpretable and transferable effects on potency, selectivity, solubility, and metabolic stability. However, ML models trained on broad chemical datasets may overestimate their ability to transfer SAR across scaffolds, targets, or assay formats. Activity cliffs—small structural changes that produce large activity differences—are particularly problematic because they contradict smooth interpolation assumptions and can cause apparently accurate global models to fail on the next analog proposed for synthesis. For this reason, ML evaluation should include series-aware validation, matched molecular pair analysis, scaffold-based splits, and temporal splits that mimic prospective project progression [41,60].

Series-based learning can help bridge this gap. Instead of treating every molecule as an independent point in chemical space, project-level models can encode R-group substitutions, matched molecular-pair transformations, shared core structures, and assay chronology [65]. Such models are better aligned with the way medicinal chemists ask questions. For example, which substituent improves potency without increasing lipophilicity? Does the para-to-meta change preserve activity? Is the current SAR transferable to a second chemo-type? By explicitly learning within-series trends and flagging extrapolation beyond a series, ML can support analog design while avoiding misleading claims of generalization [66,67].

#### 3.3.4. Scaffold Hopping: Opportunity and Risk

Scaffold hopping illustrates both the promise and danger of ML-guided molecular generation. Generative and similarity-learning models can propose structurally novel chemotypes that preserve predicted activity, potentially escaping intellectual-property constraints, improving physicochemical properties, or identifying new binding solutions [37,40]. However, novelty alone is not a medicinal chemistry success criterion. A new scaffold may lose the original binding mode, introduce new tautomeric or protonation behavior, disrupt key water-mediated interactions, alter selectivity, or become synthetically impractical. ML-driven scaffold hopping should therefore be constrained by pharmacophore hypotheses, structure-based docking or molecular dynamics where appropriate, ligand efficiency metrics, selectivity models, and explicit uncertainty estimates [57,62].

A practical safeguard is to treat scaffold hops as hypotheses requiring staged validation rather than as direct replacements for optimized series. Candidate scaffold hops should be checked for binding-mode plausibility, synthetic accessibility, novelty relative to the internal collection, similarity of key interaction features, and predicted off-target liabilities. Prospective testing should include a small set of analogs around the new core to determine whether a transferable SAR exists. Without these safeguards, generative models may produce attractive structures that score well in silico but do not support a developable medicinal chemistry series [63,64].

#### 3.3.5. Synthesizability and Route Feasibility

Synthesizability is broader than a numerical synthetic-accessibility score. In a discovery program, a proposed molecule is useful only if a feasible route exists using available building blocks, robust transformations, an acceptable protecting-group strategy, manageable stereochemical control, safe reagents, scalable purification, and reasonable costs and cycle times. ML can assist by integrating retrosynthetic analysis, reaction-feasibility prediction, building-block availability, step-count estimates, route confidence, and historical laboratory success rates. These constraints should be applied before compounds are nominated for synthesis, not after virtual optimization has produced chemically attractive but impractical structures [60,61,62].

Route feasibility also affects SAR generation. A synthetically elegant analog set can provide rapid, interpretable SAR, whereas a single difficult compound may consume resources without improving project understanding. Consequently, ML-guided design should favor makeable analog libraries that test clear medicinal chemistry hypotheses [67,68]. For example, models can propose matched substituent arrays, reagent-compatible transformations, or building-block-limited enumeration spaces, then rank compounds by expected information gain, predicted property improvement, and route confidence. This approach connects molecular generation with real laboratory constraints and reduces the risk that AI-selected compounds cannot be delivered in a useful design cycle [69].

#### 3.3.6. Developability Constraints and ADMET-by-Design

Lead optimization must also consider developability early. Potency improvement alone can drive compounds toward high molecular weight, excessive lipophilicity, poor solubility, low permeability, metabolic instability, CYP inhibition, hERG liability, transporter interactions, plasma protein binding, poor solid-state behavior, formulation challenges, or inadequate safety margins. ML-based ADMET models are valuable because they allow these liabilities to be evaluated before synthesis and can support multi-objective prioritization. However, they should be used as triage and hypothesis-generating tools rather than as substitutes for experimental ADMET because endpoint definitions, assay systems, and data quality vary substantially across sources [69,70].

A medicinal chemistry implementation should therefore use project-specific desirability functions rather than a single universal score. These functions can set acceptable ranges for potency, selectivity index, ligand efficiency, lipophilicity, polar surface area, solubility, clearance risk, permeability, and safety alerts, depending on the therapeutic hypothesis and route of administration. ML models can then identify compounds with balanced profiles, highlight which property drives risk, and suggest design changes that improve one property without damaging another. This is more consistent with candidate selection than optimizing a black-box composite score [71].

#### 3.3.7. Series-Based Active Learning and Design–Make–Test–Analyze Cycles

The strongest practical use of ML in medicinal chemistry is within iterative design–make–test–analyze cycles. New data arrive in small batches, are biased toward compounds that chemists were able and willing to synthesize, and may include conflicting potency, selectivity, solubility, and ADMET readouts [71,72]. Active learning can help by selecting compounds that maximize expected improvement, reduce model uncertainty, or test competing SAR hypotheses. Nevertheless, the acquisition function must be constrained by route feasibility, reagent availability, batch synthesis planning, assay capacity, and the need to preserve interpretable SAR. Otherwise, active learning may recommend isolated molecules that are statistically informative but operationally unattractive [73].

Series-based active learning is particularly important when several chemotypes compete. ML can estimate not only the best compound within a series but also the probability that additional analogs will improve the series enough to justify continued investment. This supports practical decisions such as expand, merge, pause, or terminate a chemical series. When combined with uncertainty estimates and prospective validation, such approaches make ML more relevant to real project governance than retrospective benchmark accuracy alone [74].

#### 3.3.8. Decision-Making Under Uncertainty

Real discovery decisions are made under uncertainty. Biological assays have variability, chemical series may shift unexpectedly, target biology can change, and models may be applied outside their training distribution. Therefore, ML outputs should be presented as calibrated probabilities, confidence intervals, applicability-domain warnings, or conformal prediction sets rather than as single deterministic scores. For compound prioritization, a medicinal chemistry team may need to know the probability that a molecule will meet a target product profile, the probability that it will be selective over a defined off-target panel, or the expected value of synthesizing a compound relative to its route cost and cycle time [74,75].

Uncertainty-aware decision frameworks can convert model predictions into practical actions: synthesize now, synthesize after route review, run a counter-screen, obtain additional ADMET data, keep as a backup, or terminate the series. This approach also improves transparency because it documents why a compound was advanced despite uncertainty or why a predicted high-scoring compound was rejected. In this way, ML becomes embedded in project-level risk management and candidate-selection logic, directly addressing the gap between computational prediction and medicinal chemistry practice [73,75].

In practical medicinal chemistry workflows, ML-guided optimization must extend beyond isolated potency prediction to address multi-parameter decision-making. Critical challenges include achieving selectivity across closely related targets, maintaining structure–activity relationship (SAR) continuity within compound series, and managing the risks associated with scaffold hopping [66,67]. In parallel, synthesizability, route feasibility, and broader developability constraints (e.g., stability, permeability, and formulation) play decisive roles in compound prioritization. Although emerging approaches such as series-based learning and uncertainty-aware prediction aim to better align ML outputs with iterative design–make–test–analyze cycles, their routine integration into real-world discovery pipelines remains limited.

To summarize these interconnected challenges and their practical implications, Table 1 outlines key medicinal chemistry issues, corresponding approaches, associated risks, and recommended safeguards for deploying ML in lead optimization campaigns [68].

### 3.4. Preclinical and Clinical Prediction

The evaluation of drug behavior in living organisms remains as one of the most complex steps that scientists face after achieving lead optimization. Traditional methods rely on animal studies to assess ADMET properties, which proves to be expensive and time-consuming [49]. Machine learning (ML) trained on toxicogenomic and pharmacokinetic datasets predict hepatotoxicity, cardiotoxicity, and blood–brain barrier permeability with higher accuracy than traditional QSAR methods [63,64].

The ADMET-AI platform provides computational toxicity screening support, which helps scientists eliminate toxic compounds before clinical testing [70]. The combination of multi-task learning models enables scientists to predict both molecular activity and safety simultaneously [71]. Nowadays, ML techniques assist in clinical trial design by identifying suitable patient populations and optimizing study parameters, improving translational success [72,73]. The integration of electronic health records with real-world data improves prediction accuracy, enabling the development of adaptive clinical trials. The acceptance of ML-based predictions by regulatory bodies depends on their ability to provide clear explanations about their decision-making processes [43,73].

### 3.5. Integration with Robotics and High-Throughput Experiments

**Figure 2:**
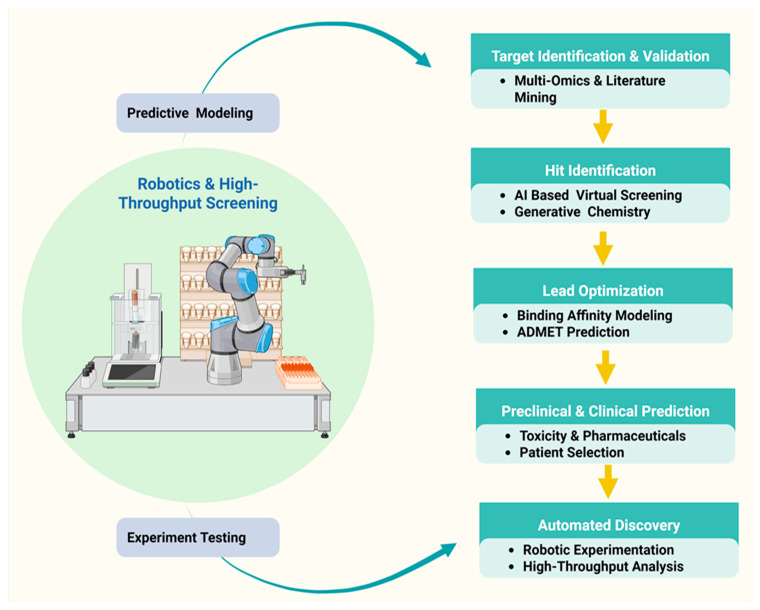
Opportunities for acceleration in ML-driven drug discovery. The process flow diagram shows how ML technology speeds up various phases of the drug discovery process. The figure shows the essential stages of intervention, which include: (1) target identification and validation through multi-omics data integration and literature mining; (2) hit identification through AI-based virtual screening and generative chemistry; (3) lead optimization through predictive modeling for binding affinity and ADMET properties; (4) preclinical and clinical prediction to predict toxicity and pharmacokinetics and patient selection; and (5) robotics and high-throughput experimentation systems work together for automated discovery processes. The diagram shows how predictive modeling and experimental testing stay connected through arrows that link these two systems (Created in BioRender. El-Tanani, M. (2026) https://BioRender.com/ui9bk2w accessed on 28 April 2026).

Machine learning (ML) integration with laboratory automation enabled the development of closed-loop discovery systems that use AI-generated hypotheses for robotic validation [76]. The automated synthesis system, together with high-throughput screening technology, provides immediate feedback to models for continuous learning and performance enhancement. The Massachusetts Institute of Technology (MIT) achieved breakthroughs in antimicrobial compound discovery through deep learning integration with robotic screening platforms, demonstrating AI-driven automation potential for therapeutic development [77,78]. The combination of ML and robotics supports adaptive experimental workflows, enhancing both speed and reproducibility (Figure 2).

Predictive modeling has greatly improved multiple phases of the drug development process. However, many ML techniques are mostly data-driven and do not completely take into consideration physical and chemical principles [79]. Another class of hybrid approaches has gained popularity in order to overcome these limitations. These physics-informed systems help enhance interpretability, generalizability, and translational robustness by directly including thermodynamic restrictions, quantum mechanical insights, and mechanistic information into model design. These technologies led to a substantial transition in drug development from statistical prediction to artificial intelligence with a mechanistic base [79,80].

### 3.6. Hybrid AI Models: Integrating Mechanism with Machine Learning

Although ML models demonstrate strong predictive performance, their reliance on data patterns can limit mechanistic understanding. To address this, hybrid approaches integrate domain knowledge from physics and chemistry into data-driven models, enhancing interpretability and robustness [81].

Physics-informed neural networks (PINNs) incorporate mathematical constraints, such as differential equations, conservation laws, thermodynamic relationships, or kinetic expressions, into the model objective so that the learned solution is discouraged from violating established physical behavior [82]. In drug discovery contexts, such constraints may be useful for drug transport, binding kinetics, enzyme kinetics, and diffusion-controlled processes, but they do not automatically guarantee mechanistic validity. The usefulness of a PINN depends on whether the imposed equations are appropriate for the biological system, whether the boundary and initial conditions are realistic, and whether the loss terms are balanced so that the model does not satisfy one constraint while ignoring another [83]. For example, diffusion-based constraints can help represent ligand concentration gradients, while thermodynamic relationships can regularize protein–ligand binding models; however, these constraints remain approximations when receptor conformational heterogeneity, solvent reorganization, protonation equilibrium, or non-equilibrium cellular processes are important. Thus, PINNs should be viewed as structured hypothesis-generating models rather than universal replacements for experimental biophysics or high-level simulation.

ML-enhanced molecular dynamics (MD) simulations combine data-driven models with conventional simulation strategies to accelerate conformational exploration, improve potential energy descriptions, identify collective variables, or prioritize adaptive sampling [84,85,86]. Their value is greatest when they help address a clearly defined sampling or force-field limitation. Nevertheless, MD remains limited by sampling sufficiency, force-field dependence, and uncertainty in initial system preparation. Short trajectories may miss slow conformational transitions, ligand unbinding events, water-network rearrangements, allosteric communication, and rare transitions that occur on microsecond-to-second timescales. Learned potentials or ML-guided sampling schemes can reduce cost, but they do not remove the need for convergence diagnostics, independent replicate trajectories, careful protonation-state assignment, ligand parameter validation, and comparison with experimental observables. In practice, uncertainty should be reported not only as model error but also as uncertainty arising from sampling, force-field choice, water and ion placement, membrane or cofactor representation, and biologically relevant system heterogeneity [86].

Hybrid docking strategies aim to combine physics-based pose generation with ML-based rescoring or ranking. These workflows can improve prioritization in virtual screening when the training domain, target class, and binding-site preparation are well aligned with the intended application [85,87]. However, docking accuracy should be decomposed into separate questions: whether the method identifies a plausible binding pose, whether it ranks active compounds above inactive compounds, and whether it predicts relative binding affinity with sufficient reliability to guide synthesis [88]. A model may place a ligand in a visually plausible pose yet fail to rank analogs correctly, and retrospective enrichment on curated benchmarks does not necessarily translate into prospective hit discovery [89,90].

A mature docking workflow must explicitly address receptor flexibility, protein protonation, ligand protonation and tautomer enumeration, stereochemistry, crystallographic water molecules, metal coordination, covalent or reversible-covalent warheads, and solvent-mediated interactions. ML rescoring functions can also learn benchmark-specific artifacts, including decoy bias, analog bias, target-family leakage, and differences between active and decoy physicochemical properties. Therefore, docking results should be evaluated using target-specific external sets, scaffold- or time-split validation where possible, enrichment metrics relevant to screening decisions, and prospective experimental confirmation rather than relying solely on retrospective benchmark performance [91,92,93,94].

To provide a structured overview of major hybrid physics–AI strategies and their practical implications in drug discovery, Table 2 summarizes the key approaches, integrated physical priors, applications, advantages, and limitations.

#### 3.6.1. Docking, ML Rescoring, and the Retrospective-to-Prospective Gap

For structure-based virtual screening, the central methodological risk is that ML can improve apparent benchmark performance without improving real discovery decisions. Receptor structures used in docking are often single, static snapshots, whereas ligand recognition may involve induced fit, side-chain rearrangement, loop motion, domain motion, ordered water displacement, or allosteric conformational selection [87]. Ensemble docking, induced-fit protocols, and MD-derived receptor conformations can partially address this limitation, but they also introduce new choices regarding ensemble size, conformer selection, and weighting. Similarly, ligand preparation is not a minor preprocessing step. Incorrect protonation, tautomer, stereochemical, or conformer enumeration can dominate docking outcomes, especially for heteroaromatic medicinal chemistry series, ionizable ligands, kinase hinge binders, metalloenzymes and solvent-exposed binding sites [93].

Solvent and electrostatics require particular caution. Docking workflows frequently treat water implicitly or remove crystallographic water molecules during preparation, yet conserved water molecules can mediate ligand binding, influence selectivity, and alter SAR interpretation. Metal coordination, halogen bonding, salt bridges, and pH-dependent charge states can also be poorly represented by generic scoring functions. ML-corrected scoring may learn patterns that improve ranking for common protein–ligand complexes, but it may fail for targets with unusual cofactors, shallow pockets, membrane exposure, covalent mechanisms, or sparse training examples. Therefore, the output of ML docking should be interpreted as a prioritization hypothesis, not as a binding free-energy measurement [81,82,83,84].

Benchmarking also requires stricter interpretation. Many docking and virtual-screening benchmarks rely on artificial decoys that may differ from active compounds in size, polarity, topology, or other easily learned properties. As a result, a classifier can appear successful by separating benchmark artifacts rather than learning true binding determinants. In addition, pose accuracy and affinity ranking are distinct tasks: a near-native pose does not guarantee correct potency ranking, and a high-scoring pose can be chemically implausible if protonation, water networks, strain, or receptor relaxation are wrong. Prospective utility should therefore be measured by enrichment of experimentally confirmed hits, hit rate, novelty, scaffold diversity, and medicinal chemistry follow-up quality, not only by retrospective ROC-AUC or docking-score correlation [94].

#### 3.6.2. ML-Enhanced MD: Sampling, Force Fields, and Uncertainty

ML-enhanced MD can support drug discovery by accelerating sampling, learning collective variables, correcting potential energy surfaces, or guiding adaptive simulations. However, the main determinant of reliability is not simulation length alone but whether relevant states and transitions have been sampled with sufficient statistical support. Binding-site hydration, loop opening, induced fit, ligand residence time, cryptic-pocket formation, and allosteric communication often involve rare events that are poorly captured by straightforward trajectories. Enhanced sampling methods may improve coverage, but biased simulations require appropriate reweighting and convergence analysis before thermodynamic or kinetic conclusions are drawn [80,94].

Force-field dependence remains a fundamental limitation. Protein, ligand, water, ion, lipid, cofactor, and metal parameters all influence conformational ensembles and binding predictions. ML potentials can improve selected energy or force estimates, but their behavior is only reliable within the chemical and conformational domain represented by the training data. This is especially important for charged ligands, zwitterions, highly flexible macrocycles, metalloproteins, post-translational modifications, membrane proteins, covalent inhibitors, and systems involving proton transfer or polarization effects. Long-time stability in a biologically complex simulation is therefore a separate requirement from low error on a small-molecule quantum-mechanical benchmark [91,92,93].

Uncertainty quantification should be incorporated into ML-MD workflows. Useful practices include replicate simulations, reporting confidence intervals for free energies or kinetic observables, ensemble or committee models for learned potentials, monitoring model disagreement during active learning, checking energy conservation and structural stability, and validating predicted conformational states against crystallography, cryo-EM, NMR, hydrogen-deuterium exchange, mutagenesis, SAR, or biophysical binding data. Without these checks, ML-enhanced MD may provide visually persuasive trajectories while still giving overconfident or non-transferable mechanistic conclusions [93,94,95].

#### 3.6.3. Practical Validation Requirements for Hybrid Physics-AI Work-Flows

Across docking, MD, and hybrid physics-AI models, the key question is whether the computational result changes a discovery decision in a reproducible and experimentally useful way. A rigorous workflow should document system preparation, protonation and tautomer assumptions, receptor conformational choices, water and ion treatment, training-domain similarity, uncertainty estimates, and prospective validation strategy. The model should also be evaluated against the decision it is intended to support: pose generation, analog ranking, hit triage, free-energy estimation, mechanism elucidation, or prioritization of compounds for synthesis [75,93].

To consolidate the methodological limitations discussed above and to define practical validation expectations for computational chemistry applications of ML, Table 3 summarizes key workflow-specific challenges and recommended evaluation strategies.

### 3.7. Integration with Quantum Mechanics

QM/MM methods are indispensable when drug–target recognition involves electronic rearrangement that cannot be represented adequately by classical force fields, such as covalent inhibition, enzymatic catalysis, charge transfer, proton transfer, metal coordination, or polarization-dominated binding [103,104]. In these simulations, a chemically active region is treated quantum mechanically while the surrounding protein, solvent, membrane, or ligand environment is described at the molecular–mechanical level. The accuracy of the result depends strongly on the definition of the QM region, treatment of the QM/MM boundary, electrostatic embedding, link-atom placement, choice of functional or wave-function method, basis set, dispersion correction, charge and spin assignment, and the extent to which relevant conformational states are sampled. Consequently, QM/MM should not be described as a uniformly high-fidelity solution; rather, it is a powerful but assumption-sensitive method whose conclusions require sensitivity analysis and comparison with experimental or higher-level theoretical evidence [103,104,105].

ML potentials and ML/MM approaches can accelerate parts of this workflow by approximating expensive quantum-mechanical energies and forces, but claims of “DFT-level accuracy” should be carefully restricted to the chemical space, reference method, and target property used for training and validation [106,107]. A potential that performs well for equilibrium geometries or neutral organic fragments may not transfer to transition states, proton-shuttling networks, charged intermediates, spin-state changes, organometallic centers, metalloenzymes, or covalent warhead chemistry. Reactive pathways are especially demanding because small errors in barrier heights can lead to large errors in predicted rates or mechanistic preference [108]. Therefore, ML potentials used for reactive drug discovery problems should be accompanied by out-of-domain tests, active-learning coverage of high-uncertainty configurations, validation against high-level quantum calculations for key stationary points, and explicit reporting of uncertainty or model disagreement [106,107,108,109].

For medicinal chemistry decision-making, the most appropriate role of QM/MM and ML potentials is often mechanistic interpretation and prioritization rather than definitive prediction [110]. These methods can help rationalize covalent inhibitor reactivity, protonation-state preferences, metal-binding geometry, ligand strain, water-mediated catalysis, or selectivity differences across homologous targets. However, they should be integrated with SAR, mutagenesis, crystallography or cryo-EM, kinetic measurements, and orthogonal biochemical data. Overstating the generality of ML potentials can be misleading. Their value depends on transparent domain-of-applicability assessment, reproducible system preparation, and prospective testing in the specific target class under study [111].

Overall, the integration of ML with quantum and physics-based methods is best framed as a route to better calibrated and more interpretable hypotheses, not as a blanket guarantee of higher accuracy [111].

## 4. Challenges and Limitations

Despite significant progress, machine learning (ML) in drug discovery faces multiple substantial obstacles, including data quality limitations, interpretability problems, generalizability challenges, regulatory hurdles, and ethical concerns [112].

### 4.1. Data Quality and Bias

The performance of machine learning (ML) models in drug discovery depends heavily on the quality and diversity of the data used to train them. If training data are incomplete, noisy, or biased, the model will generate flawed predictions. Many biochemical and pharmacological databases contain missing values, experimental noise, and biases toward known molecular targets and chemical scaffolds [113,114]. Public databases such as ChEMBL and PubChem are a great resource but contain heterogeneous assay conditions and incomplete experimental parameters, which in turn generate uncertainty in models predictions. Therefore, data curation and quality control are still necessary steps in building reliable computational models in drug discovery [115].

Limited representation of diverse chemical and biological spaces further restricts model generalizability [116]. The performance of a model can be severely affected by even small measurement errors in key variables, such as binding affinity or biological activity [117]. Improving data reliability requires systematic curation, standardization of metadata, and incorporation of uncertainty quantification strategies. These steps are essential for developing robust and interpretable ML models in drug discovery [111,118].

### 4.2. Interpretability and Explainability

A major limitation of many ML models, particularly deep learning approaches, is their lack of interpretability. ‘Black box’ deep learning models in chemistry and biology are criticized for predicting without clear explanations, making it difficult to assess their reliability and biological possibility [119,120].

The lack of transparency in ML models creates doubts about their result reliability and creates substantial barriers for regulatory approval because interpretability stands as a necessary condition for validation and approval [121]. In this context, it is important to distinguish interpretability from explainability. Interpretability refers to understanding how a model is structured and operates, whereas explainability focuses on why a specific prediction was made. Regulatory agencies increasingly recognize post hoc explanation tools such as Shapley Additive Explanations (SHAP) and attention-based methods as useful for supporting model transparency; however, these approaches are generally considered complementary rather than sufficient on their own, and they must be combined with robust validation and reproducibility to meet regulatory expectations [122]. Feature attribution methods, including SHAP and attention-based approaches, have been developed to provide insights into model predictions [120,122]. The process of model simplification for better explainability results in reduced predictive accuracy, highlighting a trade-off between accuracy and explainability [123].

### 4.3. Generalizability and Transferability

Machine learning models trained for specific chemical or biological domains fail to generalize to new molecular structures or biological targets [124]. This issue is commonly known as ‘domain shift,’ which limits model applicability in real-world scenarios. Models that perform well on training data may show reduced accuracy when applied to new molecular structures or experimental conditions. Pre-trained models and transfer-learning approaches have been introduced to address these limitations by leveraging large-scale datasets to improve robustness and adaptability [125,126]. The field faces an ongoing challenge as it lacks established methods to evaluate model reliability and generalize performance. Therapeutics Data Commons (TDC) and MoleculeNet have developed structured assessment frameworks, but their development needs to continue for establishing uniform evaluation standards [127,128,129,130].

### 4.4. Regulatory and Validation Hurdles

**Figure 3:**
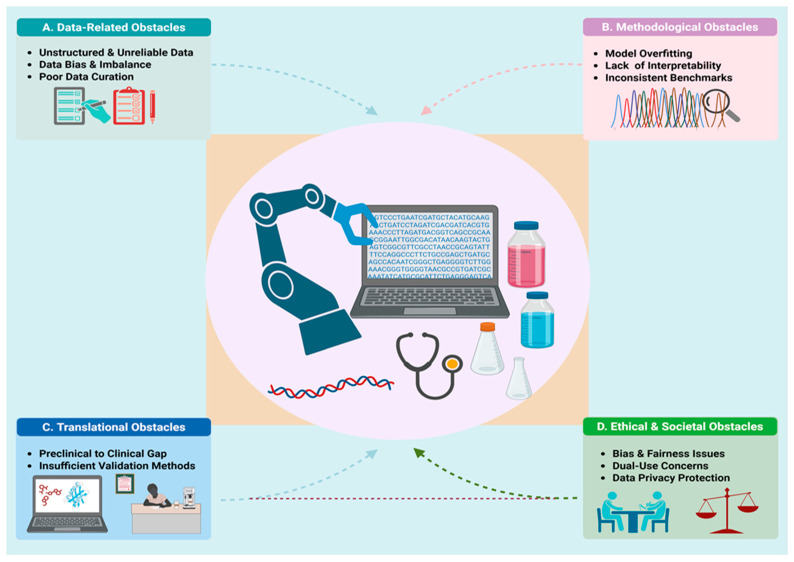
The development of machine learning-based drug discovery faces various unresolved problems which prevent its advancement. The figure presents a conceptual summary that identifies ongoing obstacles that prevent AI from becoming operational in pharmaceutical research and development. The schematic presents four primary sections, including (A) data-related obstacles due to unorganized, untrustworthy, and prejudiced datasets; (B) methodological obstacles due to model overfitting, lack of interpretability, and inconsistent benchmarking standards; (C) translational obstacles which exist between computer-based prediction systems and medical testing procedures; and (D) ethical and societal obstacles, which include bias problems, dual-use concerns, and data protection issues. Together, these elements illustrate why robust data curation, transparent modeling, and ethical governance remain prerequisites for reliable ML-driven innovation (Created in BioRender. El-Tanani, M. (2026) https://BioRender.com/ui9bk2w accessed on 28 April 2026).

The U.S. Food and Drug Administration (FDA) and European Medicines Agency (EMA) support AI implementation in drug development, yet they demand complete transparency, reproducibility, and traceability in all algorithmic operations [131]. The current regulatory requirements demand that ML-based predictions used for preclinical or clinical choices need to be both traceable and explainable to allow independent verification and scientific validation [132]. The field lacks a standardized method to assess ML algorithms used in drug discovery operations. The development of standardized validation protocols, continuous-learning oversight systems, and GMLP framework integration will establish accountability, reliability, and regulatory compliance throughout the pharmaceutical development process [133] (Figure 3).

### 4.5. Ethical, Legal, and Security Considerations

The implementation of machine learning (ML) in drug discovery creates new ethical problems, legal issues, and security risks, which go beyond technical aspects [134]. Generative models capable of designing new compounds could potentially generate harmful or dual-use compounds, posing significant security risks. To mitigate these risks, emerging strategies include red-teaming approaches to systematically probe model vulnerabilities, in silico toxicity and dual-use screening pipelines to filter generated compounds, and controlled access frameworks that restrict model deployment and usage. Together, these measures aim to balance innovation with responsible governance and reduce the potential for misuse [135,136].

The combination of patient data with predictive models increases the risk of security breaches that threaten personal information protection [137]. Additionally, existing legal frameworks do not clearly address issues related to intellectual property and authorship for AI-generated compounds [138]. The development of AI-enhanced therapeutics needs to occur under rules that protect patient information while ensuring medical treatment and limiting algorithmic discrimination in healthcare choices [139].

### 4.6. Cultural and Organizational Barriers

**Figure 4:**
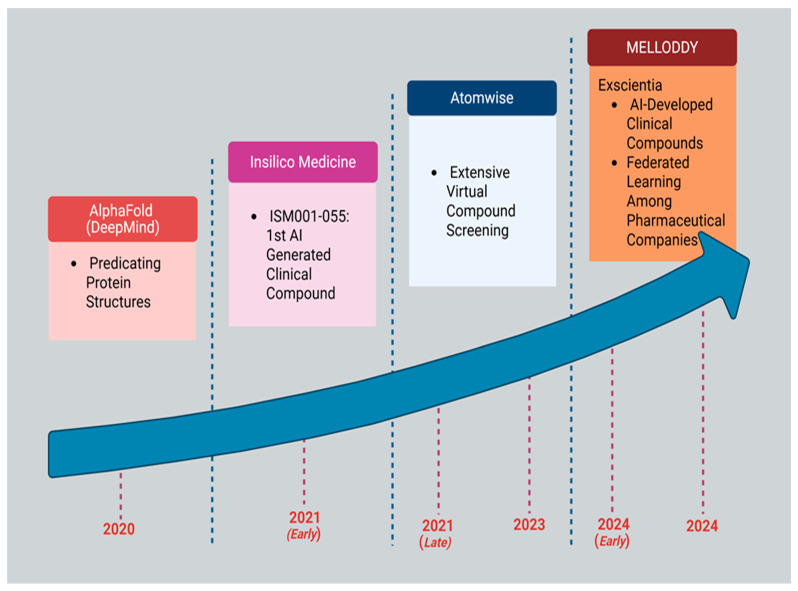
Case studies and success stories in AI-driven drug discovery. The infographic shows a timeline demonstrating essential machine learning discoveries during actual drug development research. The research includes AlphaFold (DeepMind), which uses DeepMind to predict protein structures; Insilico Medicine’s ISM001-055 as the first AI-generated compound for human clinical testing; Atomwise’s AtomNet system, which performs extensive virtual compound screening; Halicin as a deep-learning-based antibiotic with a distinctive chemical structure; Exscientia, which uses artificial intelligence to develop medicinal compounds for clinical use; and the MELLODDY consortium, which proves that pharmaceutical companies can use federated learning to work together. These cases show how AI technology reduces discovery times while scientists discover new chemical compounds and scientists can work together through data analysis (Created in BioRender. El-Tanani, M. (2026) https://BioRender.com/ui9bk2w accessed on 28 April 2026).

Adoption of ML in pharmaceutical research is influenced not only by technical challenges but also by organizational and cultural factors. Traditional drug discovery workflows rely heavily on expert-driven decision-making, so integrating ML requires shifts in both infrastructure and mindset [140]. The implementation of ML systems faces challenges due to unexplained systems and no standardized method to verify performance [130]. Building confidence in ML systems will require collaborative efforts, improved training, and consistent demonstration of reproducible results [141] (Figure 4).

### 4.7. ML Failures in Drug Discovery

Despite rapid advancements, ML applications in drug discovery face several practical limitations that highlight the need for careful evaluation. A common issue is overfitting to biased datasets, where models capture patterns specific to well-studied targets or chemical scaffolds but fail to generalize to new data [142,143]. Differences between public benchmark datasets and proprietary industry data further contribute to variability in model performance [144]. Reproducibility remains another concern, echoing challenges previously observed in QSAR modeling. Inconsistent data curation, inadequate validation strategies, and incomplete reporting have led to difficulties in reproducing published results [145]. These issues emphasize the need for proper reporting standards and consistent benchmarks.

Generative models introduce additional challenges. While they can design novel compounds with desirable predicted properties, many generated molecules may be chemically unstable or difficult to synthesize. In some cases, proposed structures violate fundamental chemical principles or require impractical synthetic routes [146].

Although ML accelerates candidate identification, it does not eliminate the inherent complexity of biological systems. Compounds identified through ML must still undergo rigorous experimental and clinical validation, and current evidence demonstrating improved clinical success rates remains limited [147].

Additional concerns include data overlap between training and test sets, benchmark leakage, and inadequate cross-validation due to improper data partitioning strategies. To mitigate these issues, best practices increasingly emphasize the use of scaffold-based splits, which separate structurally distinct chemical scaffolds between training and test sets, as well as temporal splits that reflect real-world prospective discovery settings. These strategies provide more realistic estimates of model generalization and reduce the risk of benchmark leakage compared to random data partitioning [148,149]. Addressing these issues, external validation, prospective validation, and clinical validation are required to confirm the computational findings. The translational impact of computational advances can be ensured only if we adequately account for and transparently report the issues of reproducibility, data bias, and clinical relevance [150].

A medicinal-chemistry-specific failure mode occurs when an ML model appears successful on benchmark potency prediction but fails to improve real lead optimization decisions. Examples include recommending compounds that are outside the active series without adequate SAR rationale, proposing scaffold hops without a plausible binding mode, improving predicted potency while worsening selectivity or developability, or selecting molecules that cannot be synthesized within a project-relevant cycle. These failures are often invisible in random train–test splits and become apparent only in scaffold-based, temporal, and prospective validation settings that reflect how project teams actually nominate compounds [138,139,140].

## 5. Future Directions and Translational Outlook

The adoption of machine learning (ML) technology in drug discovery has reached a critical point as it moves from exploratory testing to potential industrial adoption [151]. The achievement of ML for long term impact depends on advancements in five essential areas, which include model generalizability, multimodal data integration, interpretability, automation, and human–AI collaboration [152].

### 5.1. Foundation and Multimodal Models

The recent development of foundation models trained on extensive chemical, biological, and textual data has revolutionized molecular science [152]. Differentiating between task-specific models and pretrained foundation models is crucial for showcasing how AI is transforming drug discovery. Task-specific models are trained on small, labeled datasets (10^3^–10^5^ molecules) to predict specific tasks (binding affinity, toxicity, etc.), with 10^4^–10^7^ parameters, which can be trained quickly but are not easily transferable beyond their distribution of training data [153,154].

On the other hand, pretrained foundation models are large (10^7^–10^9^ parameters) and are trained on millions to billions of chemical or protein sequences using self-supervised learning. Pretrained foundation models learn large, general representations of chemical and biological interactions, enabling them to adapt multiple downstream tasks, including molecular property prediction, drug–target interaction analysis, and compound generation [155].

Multimodal LLM-based biomedical agents will be the next generation of AI for drug discovery. These models can integrate multiple types of input, including omics datasets, structural biology data, genome data, and even the scientific literature, within a unified framework [156]. As we scale up the parameters and data, the question of computational cost and environmental impact become increasingly relevant. Training large models requires a high-performance computing capacity that requires high energy and carbon emissions. Thus, to maintain scientific rigor and ecological responsibility for foundation-scale AI advancements, responsible model development must incorporate energy-efficient structures, training paradigms, and computing [157,158].

The training of MolBERT, ChemGPT, and ESMFold models on millions of molecular structures and protein sequences has assisted scientists in developing comprehensive cellular models that predict disease mechanisms and the effects of therapies [159,160]. The development of these large models requires significant computational resources and appropriate data management systems to avoid a single organization from monopolizing both data and model ownership and to allow model transparency for global scientific access [161].

### 5.2. Human–AI Collaboration

AI systems function as cognitive collaborators that enhance human scientific abilities and creative thinking rather than replacing researchers during discovery work [162]. Modern drug discovery platforms allow researchers to explore the chemical space, evaluate hypotheses, and optimize compounds through interactive, AI-assisted workflows [163]. The advancement of explainable and interactive systems will further support real-time collaboration between researchers and AI models. Such approaches enable iterative refinement of hypotheses and improve decision-making by providing interpretable insights [164].

### 5.3. Active Learning and Autonomous Discovery

The integration of active-learning systems with high-throughput experimentation and automation enables self-adaptive discovery processes that enhance predictive accuracy through continuous feedback loops [165]. Autonomous laboratories uniting robotics with machine learning and cloud-based analytics have become revolutionary platforms that produce experimental data in real time to speed up scientific progress. While these systems offer significant potential, their effectiveness depends on robust data quality control, safety protocols, and mechanisms to prevent model drift [166].

### 5.4. Open Science, Data Sharing, and Collaboration

The success of ML-based drug discovery requires open data platforms that offer standardized benchmarks to promote scientific transparency [165]. The Open Reaction Database, Therapeutics Data Commons, and AlphaFold Protein Structure Database serve as examples of collaborative platforms that provide equal access to molecular information to accelerate scientific progress between institutions [164,166]. The development of this ecosystem requires academic institutions, biotechnology companies, and regulatory agencies to work together through worldwide consortia for creating standardized protocols that support open data sharing and enable AI-driven discovery validation [167,168]. The advancement of this system requires proper management of patient information security, intellectual property protection, and cybersecurity measures to achieve ethical and protected data exchange.

### 5.5. Responsible and Sustainable AI

The operation of AI-based research facilities faces growing sustainability concerns because model training requires massive computational resources, which generate substantial environmental carbon emissions. The future development of AI systems will focus on green AI principles, which include designing energy-efficient systems using low-precision calculations and implementing carbon offset programs [167]. The practice of responsible AI requires more than technical improvements; it must provide equal access to AI-based medical solutions for all population groups [168].

### 5.6. Toward Digital Twins and Precision Pharmacology

The development of digital twins through AI-based biomedical innovation aims to create virtual human models for predicting disease evolution and drug reactions [169]. These systems have the potential to predict disease progression and treatment response, supporting personalized therapeutic strategies and improved clinical trial design. The combination of digital twin systems with extensive foundation models will create individualized personalized medicine, which will revolutionize drug development and precision medicine [170].

### 5.7. Critical Analytical Framework

In ML-driven drug discovery, evidence should therefore be interpreted across a hierarchy of maturity rather than as a single category of “AI success.” At the lowest level are retrospective benchmark studies that compare algorithms on curated datasets. These are useful for method development but are vulnerable to dataset bias, train-test leakage, decoy artifacts, and overly optimistic random splits. A stronger level of evidence is obtained when models are evaluated on external datasets, scaffold-disjointed or temporally separated test sets, and prospectively registered performance criteria [103,119]. The most persuasive evidence is prospective experimental validation in which model-nominated compounds, targets, or hypotheses are tested under predefined assay conditions, followed by demonstration that the model improved real project decisions, such as reducing design–make–test–analyze cycles, improving selectivity or ADMET balance, or increasing the probability of candidate progression [138,139,140].

To provide a structured framework for evaluating the strength of evidence and common sources of overstatement in ML-driven drug discovery, Table 4 summarizes the key claim categories, minimum validation requirements, current confidence levels, and typical interpretational pitfalls.

This framework leads to four evaluative questions that should be applied consistently across target discovery, virtual screening, generative chemistry, ADMET prediction, docking, MD, QM/MM, foundation models, and autonomous platforms: (i) what decision is the ML model intended to support; (ii) what comparator represents current best practice; (iii) what type of validation demonstrates generalization beyond the training domain; and (iv) what experimental or translational outcome shows that the prediction changed a discovery decision rather than merely improving a retrospective metric [129,130,131,132,133,134].

Using this hierarchy, the strongest current value of ML is found in constrained, data-rich tasks where the prediction target is well defined and where external validation is feasible. Examples include molecular property prediction for standardized endpoints, structure-informed prioritization when the target biology is well characterized, ADMET triage for large compound libraries, and protein-structure-enabled hypothesis generation. Even in these settings, the claim should usually be framed as decision support rather than autonomous discovery because the final project decision still depends on medicinal chemistry judgment, assay context, synthetic feasibility, and biological validation [128].

By contrast, evidence remains preliminary for broad de novo molecular generation, general-purpose biomedical agents, digital twins for individual patients, and fully autonomous discovery platforms [146,147,148,149]. These approaches are scientifically important, but many reports demonstrate feasibility within selected examples rather than reproducible improvement across independent discovery campaigns. Claims in these areas should be qualified by the level of experimental confirmation, the number of compounds synthesized and tested, the attrition rate from generated ideas to validated hits, and whether the model improved a decision that would otherwise have been made by conventional computational or medicinal chemistry practice [169,170].

Acceptable validation practice should therefore go beyond random train–test splits and retrospective benchmark tables. For molecular models, validation should include chemically meaningful partitioning, ideally scaffold-based or temporal splits, external datasets where possible, calibration of predicted probabilities or error intervals, explicit applicability-domain assessment, and reporting of negative or failed predictions. For prospective studies, authors should predefine hit-selection criteria, assay conditions, baseline methods, and success metrics; report all nominated compounds or hypotheses where feasible; and distinguish model performance from downstream human filtering, synthetic triage, or assay rescue [147,148,149,150].

A critical synthesis should also identify overstated language. “Acceleration” should be reserved for studies showing fewer experimental cycles, faster validated hit identification, or improved candidate quality relative to a comparator, not merely faster in silico scoring [130,131,132,133,134]. Novelty should mean more than structural dissimilarity; it should include chemical plausibility, synthetic accessibility, and evidence that the molecule is not simply an artifact of the training distribution. “Clinical translation” should not be inferred from computational prioritization or platform announcements unless supported by preclinical or clinical progression data. Similarly, “end-to-end AI discovery” should be used cautiously when target selection, assay design, compound synthesis, or candidate nomination remain heavily dependent on human expert intervention [169,170,171,172,173].

This distinction is especially important because proof-of-concept studies, platform case reports, benchmark improvements, and translational outcomes answer different scientific questions. A proof-of-concept study asks whether an approach can work under controlled conditions. A platform claim asks whether a workflow can generate plausible outputs at scale [173]. A methodological benchmark asks whether one model outperforms another under a defined data split. A translational outcome asks whether the approach improves a real discovery decision or development trajectory. These categories should not be placed on the same evidentiary level. The most reliable reviews and benchmarks will explicitly state which level of evidence each cited study provides and avoid presenting methodological promise as established therapeutic impact [171,172].

Within this framework, the impact of machine learning (ML) in drug discovery remains uneven across application domains. Robust value has been demonstrated in well-defined, data-rich tasks, such as molecular property prediction and protein structure modeling, where standardized benchmarks and external validation are increasingly available. By contrast, areas such as de novo molecular generation and multimodal foundation models remain largely at the proof-of-concept stage, with limited prospective validation and uncertain translational outcomes [169].

A key limitation across the field is inconsistency in validation practices. Over-reliance on random data splits, limited external validation, and potential benchmark leakage can lead to overly optimistic performance estimates. Best practices, including scaffold-based and temporal validation strategies, remain underutilized but are essential for assessing real-world generalizability. Furthermore, many reported advances focus on predictive accuracy without sufficient consideration of experimental feasibility, synthetic accessibility, or clinical relevance [172].

Importantly, claims regarding fully autonomous or “end-to-end” AI-driven drug discovery systems should be interpreted with caution. While integrated platforms show promise, current evidence suggests that ML functions most effectively as an augmentative tool within human-guided workflows rather than as a replacement for domain expertise [173]. Bridging the gap between computational predictions and clinical success will require rigorous prospective validation, standardized benchmarking, and closer integration with experimental and regulatory frameworks. From a computational chemistry perspective, the same caution applies to hybrid physics-AI claims: retrospective improvements in docking, MD, or learned-potential benchmarks should not be equated with prospective enrichment, accurate kinetics, reliable free energies, or transferable chemical reactivity. The most credible studies will report domain-of-applicability limits, uncertainty, system-preparation assumptions, and experimental validation tied to the intended discovery decision [174].

For future evaluations, medicinal chemistry relevance should be reported explicitly. Reviews and benchmarks should ask whether an ML method improves compound-series prioritization, selectivity windows, synthetic tractability, and candidate-quality decisions, not only whether it improves retrospective AUC, RMSE, or enrichment metrics. Prospective studies should report how many model-nominated compounds were synthesized, how many routes were feasible, whether the expected SAR trend was observed, and whether uncertainty estimates changed project decisions. These criteria would make the translational value of ML more transparent to medicinal chemists, pharmacologists, and regulators [172,173,174,175,176].

## 6. Conclusions

Machine learning (ML) has evolved from a supporting computational tool into a central component of modern drug discovery, reshaping how targets are identified, compounds are designed, and decisions are made across the development pipeline. High-profile advances, including protein structure prediction, generative chemistry, and AI-assisted screening, demonstrate the potential of ML to accelerate discovery and expand accessible chemical space. However, despite these achievements, the field remains in a transitional phase in which methodological progress has outpaced consistent demonstration of translational impact.

A central theme of this review is that the value of ML in drug discovery is not determined solely by predictive accuracy but by its ability to improve real medicinal chemistry decisions. Effective deployment requires models that operate within the constraints of compound series, selectivity requirements, synthetic feasibility, and multi-parameter optimization. As highlighted throughout this work, challenges such as SAR discontinuities, scaffold hopping risks, data bias, and limited applicability domains continue to restrict generalizability. In this context, ML should be viewed primarily as a decision-support framework that augments, rather than replaces, expert-driven design.

The integration of physics-based methods with data-driven models represents a promising direction toward improved interpretability and mechanistic grounding. Hybrid approaches, including ML-enhanced molecular dynamics, AI-corrected docking, and QM/MM–ML workflows, offer opportunities to bridge statistical prediction with physical realism. Nevertheless, these approaches remain sensitive to system preparation, sampling limitations, and domain-specific assumptions, reinforcing the need for rigorous validation and uncertainty quantification. Across all applications, the distinction between retrospective performance and prospective utility remains critical, and claims of acceleration or accuracy must be supported by experimentally validated outcomes.

Looking forward, the most meaningful progress will depend on the adoption of standardized validation frameworks, increased use of prospective and externally validated studies, and tighter integration of ML within iterative design–make–test–analyze cycles. Advances in multimodal foundation models, active learning, and autonomous experimentation may further enhance discovery efficiency, but their impact will depend on transparency, reproducibility, and alignment with real-world constraints. Equally important are considerations of data governance, sustainability, and equitable access, which will shape the responsible deployment of AI in pharmaceutical research.

Machine learning holds substantial promise to transform drug discovery, but its long-term impact will depend on disciplined application, critical evaluation, and close integration with experimental science. Rather than replacing established practices, ML is most effective when embedded within a rigorous, hypothesis-driven framework that prioritizes decision quality, reproducibility, and translational relevance.

## Figures and Tables

**Table 1:** Medicinal chemistry-specific issues that ML models must address during lead optimization.

Medicinal Chemistry Issue	How ML Can Help	Practical Risk	Suggested Safeguards/Use in Campaigns	Ref.
Selectivity optimization	Multi-task target-family models, off-target panel prediction, and active learning for counter-screening.	Sparse and biased counter-screen data may hide anti-target liabilities.	Model selectivity margins, report uncertainty, and trigger focused counter-screens.	[60,61,62]
SAR continuity and transferability	Series-based models, matched molecular pairs, R-group encodings, scaffold and temporal validation.	Global models may miss activity cliffs and exaggerate transfer across scaffolds.	Use series-aware validation and flag compounds outside the applicability domain.	[37,41]
Scaffold hopping	Generative and similarity-learning models can propose alternative chemotypes.	New scaffolds may lose binding mode, selectivity, SAR interpretability, or route feasibility.	Require pharmacophore/structure checks, uncertainty estimates, and prospective analog follow-up.	[57,62]
Synthesizability and route feasibility	Retrosynthesis, reaction-feasibility models, building-block filters, and route-confidence scoring.	Synthetic-accessibility scores may not reflect real cycle time, stereochemistry, scale-up, or purification.	Constrain design by route feasibility and prioritize makeable analog sets.	[66,67,68]
Developability constraints	Multi-objective ADMET and physicochemical-property prediction.	Single composite scores may hide solubility, permeability, metabolic, or safety liabilities.	Use project-specific desirability functions and experimental ADMET confirmation.	[69,70,71,72,73]
Decision-making under uncertainty	Bayesian ensembles, calibrated probabilities, applicability-domain alerts, and conformal prediction.	Deterministic rankings can overstate confidence and mislead candidate selection.	Translate uncertainty into actions: make, test, counter-screen, hold, or terminate.	[74,75]

**Table 2:** Hybrid physics–AI integration strategies.

Approach	Physical Prior Integrated	Application	Advantage	Limitation	Ref.
Physics-Informed Neural Networks (PINNs)	PDE constraints, thermodynamics	Binding kinetics	Mechanistic interpretability	Sensitive to loss-term weighting, boundary conditions, and misspecified physical constraints	[90,91]
ML-Enhanced Molecular Dynamics	Force-field correction	Protein–ligand dynamics	Improved realism	Sampling convergence, force-field dependence, rare-event kinetics, and uncertainty propagation	[95,96]
AI-Corrected Docking	Scoring function recalibration	Virtual screening	Reduced false positives	Receptor flexibility, ligand microstates, solvent effects, decoy bias, and retrospective-to-prospective gaps	[96,97,98,99]
QM/MM + ML	Quantum mechanical priors	Reaction modeling	High chemical fidelity	QM-region definition, boundary artifacts, level-of-theory dependence, and limited reactive transferability	[100,101]
Learned Force Fields (ANI, NequIP)	Symmetry, energy conservation	Molecular simulation	High accuracy	Out-of-domain extrapolation, reactive events, metal centers, and long-timescale stability	[100,101,102]

**Table 3:** Methodological limitations and validation expectations for computational chemistry applications of ML.

Workflow	Key Limitations Requiring Explicit Treatment	Validation Expectations	Ref.
Docking/ML rescoring	Receptor flexibility; protonation, tautomer and stereochemical enumeration; water and solvent effects; metal coordination; decoy and analog bias; pose accuracy versus affinity ranking; retrospective benchmarks versus prospective enrichment.	Use careful protein and ligand preparation, ensemble or induced-fit strategies when justified, water/metal-aware protocols, target-specific external validation, enrichment metrics relevant to screening, and experimental hit confirmation.	[83,84,85,86]
ML-enhanced MD	Sampling insufficiency; force-field and system-preparation dependence; rare-event kinetics; limited treatment of slow conformational changes; uncertainty from water, ion, membrane, cofactor, and ligand parameters.	Use replicate trajectories, convergence diagnostics, enhanced-sampling reweighting where appropriate, uncertainty intervals, comparison with structural/biophysical data, and explicit reporting of simulation assumptions.	[91,92,93]
QM/MM integration	Choice of QM region; boundary and link-atom artifacts; electrostatic embedding; level-of-theory and basis-set dependence; charge, spin and protonation-state ambiguity; limited sampling of reactive configurations.	Test QM-region and method sensitivity, compare barriers and reaction energies across levels of theory where feasible, validate against structural, kinetic, spectroscopic, or mutational evidence, and report uncertainty qualitatively or quantitatively.	[95,96,97,98,99,100,101]
ML potentials/learned force fields	Training-domain dependence; out-of-distribution extrapolation; reactive intermediates; transition states; proton transfer; charge transfer; metal coordination; long-time stability in heterogeneous biomolecular systems.	Use active learning or model-disagreement monitoring, external quantum-mechanical test sets, high-level reference calculations for critical states, stability checks in biomolecular simulations, and restrained claims about DFT-level accuracy.	[96,103]

**Table 4:** Critical analytical framework for evaluating ML in drug discovery.

Evidence/Claim Category	Minimum Acceptable Support	Current Confidence Level	Typical Overstatement to Avoid	Ref.
Retrospective benchmark performance	Curated data provenance, leakage checks, scaffold/temporal splits, uncertainty and calibration reporting, and comparison with strong non-ML baselines.	Useful for method ranking but insufficient for translational claims.	Equating high AUC/RMSE improvement with discovery acceleration or clinical relevance.	[128,129]
Virtual screening and docking	Prospective enrichment or experimental hit confirmation; explicit treatment of receptor preparation, protonation/tautomer states, solvent, decoy bias, and pose/affinity separation.	Moderate when prospectively validated; limited when only retrospective.	Claiming “accurate binding prediction” from retrospective docking scores alone.	[89,90,91,92,93]
ADMET and property prediction	External validation on assay-consistent datasets, applicability-domain analysis, endpoint-specific uncertainty, and evidence that predictions guide compound prioritization.	Relatively robust for data-rich endpoints; weaker for sparse or context-dependent toxicities.	Assuming broad safety prediction from narrow endpoint performance.	[128,129]
Generative molecular design	Chemical validity, novelty relative to training data, synthesizability, route feasibility, experimentally confirmed activity, and attrition reporting for generated compounds.	Promising but often preliminary.	Describing generated structures as drug candidates before synthesis and biological validation.	[146,147,148,149]
Foundation models and AI agents	Task-specific external validation, ablation against smaller models, compute/resource reporting, uncertainty handling, and human-in-the-loop evaluation.	Emerging; strong claims require prospective demonstration.	Presenting broad language-model capability as end-to-end discovery competence.	[151,152,153,154,155,156,157,158,159,160]
Autonomous or closed-loop discovery	Predefined objectives, robotic/assay quality control, reproducible feedback cycles, negative-result reporting, and comparison with expert-guided workflows.	Potentially high value where experimentally demonstrated.	Claiming autonomy when human triage, synthesis choices, or assay design remain decisive.	[164,165]
Translational or clinical impact	Evidence of improved candidate quality, reduced cycle time, better trial selection, regulatory-grade traceability, or clinical outcome relevance.	Strong only when linked to prospective development outcomes.	Treating platform announcements or single case studies as proof of generalizable clinical impact.	[170,171,172]

## Data Availability

No new data were created or analyzed in this study.

## References

[1] Husnain A., Rasool S., Saeed A., Hussain H.K. (2023). Revolutionizing pharmaceutical research: Harnessing machine learning for a paradigm shift in drug discovery. Int. J. Multidiscip. Sci. Arts.

[2] Hakami M.A. (2024). Harnessing machine learning potential for personalised drug design and overcoming drug resistance. J. Drug Target..

[3] Thakur Z., Bansal L., Mehta P.K. (2026). Harnessing artificial intelligence and machine learning to accelerate structure-based drug discovery for tuberculosis. Future Med. Chem..

[4] Dangeti A., Bynagari D.G., Vydani K. (2023). Revolutionizing drug formulation: Harnessing artificial intelligence and machine learning for enhanced stability, formulation optimization, and accelerated development. Int. J. Pharm. Sci. Med..

[5] Singh S., Shingatgeri V., Srivastava P. (2024). Revolutionizing new drug discovery: Harnessing AI and machine learning to overcome traditional challenges and accelerate targeted therapies. Artif. Intell. Health.

[6] Verma A., Awasthi A. (2024). Revolutionizing drug discovery: The role of artificial intelligence and machine learning. Curr. Pharm. Des..

[7] Hashemi S., Vosough P., Taghizadeh S., Savardashtaki A. (2024). Therapeutic peptide development revolutionized: Harnessing the power of artificial intelligence for drug discovery. Heliyon.

[8] Pradhan T., Gupta O., Chawla G. (2024). The future of ChatGPT in medicinal chemistry: Harnessing AI for accelerated drug discovery. ChemistrySelect.

[9] Agrahari V., Choonara Y.E., Mosharraf M., Patel S.K., Zhang F. (2024). The role of artificial intelligence and machine learning in accelerating the discovery and development of nanomedicine. Pharm. Res..

[10] Ocana A., Pandiella A., Privat C., Bravo I., Luengo-Oroz M., Amir E., Gyorffy B. (2025). Integrating artificial intelligence in drug discovery and early drug development: A transformative approach. Biomark. Res..

[11] Terranova N., Renard D., Shahin M.H., Menon S., Cao Y., Hop C.E., Hayes S., Madrasi K., Stodtmann S., Tensfeldt T. (2024). Artificial intelligence for quantitative modeling in drug discovery and development: An innovation and quality consortium perspective on use cases and best practices. Clin. Pharmacol. Ther..

[12] Liu W., Pang P.D., Wu C.A., Tagle D., Wu J.C. (2026). New approach methodologies for drug discovery. Cell.

[13] Fu C. (2025). The future of pharmaceuticals: Artificial intelligence in drug discovery and development. Adv. Drug Deliv. Rev..

[14] Wan Z., Sun X., Li Y., Chu T., Hao X., Cao Y., Zhang P. (2025). Applications of artificial intelligence in drug repurposing. Adv. Sci..

[15] Hays H., Richardson W.J. (2026). A Survey of LLMs in Drug Discovery and Precision Medicine. SSRN.

[16] Wassermann A.M., Bajorath J. (2011). BindingDB and ChEMBL: Online compound databases for drug discovery. Expert. Opin. Drug Discov..

[17] Marbán-González A., Ramírez-Cid V., Cristóbal-Ramírez A., Medina-Franco J.L. (2025). Exploiting PubChem and other public databases for virtual screening in 2025: What are the latest trends? Expert Opin. Drug Discov..

[18] Gaulton A., Bellis L.J., Bento A.P., Chambers J., Davies M., Hersey A., Overington J.P. (2012). ChEMBL: A large-scale bioactivity database for drug discovery. Nucleic Acids Res..

[19] Gilson M.K., Liu T., Baitaluk M., Nicola G., Hwang L., Chong J. (2016). BindingDB in 2015: A public database for medicinal chemistry, computational chemistry and systems pharmacology. Nucleic Acids Res..

[20] Kim S., Bolton E.E. (2024). PubChem: A large-scale public chemical database for drug discovery. Open Access Databases and Datasets for Drug Discovery.

[21] Hunter F.M.I., Ioannidis H., Bento A.P., Bosc N., Corbett S., Felix E., Magarinos M.P., Manners E., Smit I.A., de Veij M. (2025). Drug and Clinical Candidate Drug Data in ChEMBL. J. Med. Chem..

[22] Alizadehsani R., Oyelere S.S., Hussain S., Jagatheesaperumal S.K., Calixto R.R., Rahouti M., Roshanzamir M., De Albu-querque V.H.C. (2024). Explainable artificial intelligence for drug discovery and development: A comprehensive survey. IEEE Access.

[23] Mghwary A.E., Hassan R.A., Halim P.A., Abdelhameid M.K. (2025). Advances in structural identification of some thieno [2, 3-d] pyrimidine scaffolds as antitumor molecules: Synthetic approaches and control programmed cancer cell death potential. Bioorganic Chem..

[24] Sayed M.T.M., Hassan R.A., Halim P.A., El-Ansary A.K. (2023). Recent updates on thienopyrimidine derivatives as anticancer agents. Med. Chem. Res..

[25] Ali E.M., Abdel-Maksoud M.S., Oh C.H. (2019). Thieno [2, 3-d] pyrimidine as a promising scaffold in medicinal chemistry: Recent advances. Bioorganic Med. Chem..

[26] Nag S., Baidya A.T., Mandal A., Mathew A.T., Das B., Devi B., Kumar R. (2022). Deep learning tools for advancing drug discovery and development. 3 Biotech..

[27] Jiménez-Luna J., Grisoni F., Weskamp N., Schneider G. (2021). Artificial intelligence in drug discovery: Recent advances and future perspectives. Expert. Opin. Drug Discov..

[28] Ali N., Hanif N., Khan H.A., Waseem M.A., Saeed A., Zakir S., Khan A., Aamir M., Ali A., Ali A. (2025). Deep learning and artificial intelligence for drug discovery, application, challenge, and future perspectives. Discov. Appl. Sci..

[29] Nissan N., Allen M.C., Sabatino D., Biggar K.K. (2024). Future perspective: Harnessing the power of artificial intelligence in the generation of new peptide drugs. Biomolecules.

[30] Athul H., Shaji S., Shabik K., Joseph A. (2024). Harnessing the power of drug repurposing: A promising strategy for drug discovery and development. Natl. J. Pharmacol. Ther..

[31] Hasanzad M., Nosrati M., Khatami F., Rahmani P., Sarhangi N., Nikfar S., Abdollahi M. (2024). Drug discovery in the context of precision medicine and artificial intelligence. Expert. Rev. Precis. Med. Drug Dev..

[32] Askr H., Elgeldawi E., Aboul Ella H., Elshaier Y.A., Gomaa M.M., Hassanien A.E. (2023). Deep learning in drug discovery: An integrative review and future challenges. Artif. Intell. Rev..

[33] Tunduny T., Shibwabo B. (2026). Explainable AI Approaches in Federated Learning: Systematic Review. JMIR AI.

[34] Qadri Y.A., Shaikh S., Ahmad K., Choi I., Kim S.W., Vasilakos A.V. (2025). Explainable artificial intelligence: A perspective on drug discovery. Pharmaceutics.

[35] Chandrasekaran S.N., Ceulemans H., Boyd J.D., Carpenter A.E. (2021). Image-based profiling for drug discovery: Due for a machine-learning upgrade?. Nat. Rev. Drug Discov..

[36] Patel L., Shukla T., Huang X., Ussery D.W., Wang S. (2020). Machine learning methods in drug discovery. Molecules.

[37] Turzo S.B.A., Hantz E.R., Lindert S. (2022). Applications of machine learning in computer-aided drug discovery. QRB Discov..

[38] Hasselgren C., Oprea T.I. (2024). Artificial intelligence for drug discovery: Are we there yet?. Annu. Rev. Pharmacol. Toxicol..

[39] Stephenson N., Shane E., Chase J., Rowland J., Ries D., Justice N., Cao R. (2019). Survey of machine learning techniques in drug discovery. Curr. Drug Metab..

[40] Naik R.R., Shakya A.K., Aladwan S.M., El-Tanani M. (2022). Kinase inhibitors as potential therapeutic agents in the treatment of COVID-19. Front. Pharmacol..

[41] vanTilborg D., Brinkmann H., Criscuolo E., Rossen L., Özçelik R., Grisoni F. (2024). Deep learning for low-data drug discovery: Hurdles and opportunities. Curr. Opin. Struct. Biol..

[42] Gupta R., Srivastava D., Sahu M., Tiwari S., Ambasta R.K., Kumar P. (2021). Artificial intelligence to deep learning: Machine intelligence approach for drug discovery. Mol. Divers..

[43] Patel V., Shah M. (2022). Artificial intelligence and machine learning in drug discovery and development. Intell. Med..

[44] Kolluri S., Lin J., Liu R., Zhang Y., Zhang W. (2022). Machine Learning and Artificial Intelligence in Pharmaceutical Research and Development: A Review: Machine Learning and Artificial Intelligence in Pharmaceutical R&D. AAPS J..

[45] Hinkson I.V., Madej B., Stahlberg E.A. (2020). Accelerating therapeutics for opportunities in medicine: A paradigm shift in drug discovery. Front. Pharmacol..

[46] Santa Maria J.P., Wang Y., Camargo L.M. (2023). Perspective on the challenges and opportunities of accelerating drug discovery with artificial intelligence. Front. Bioinform..

[47] Bergström F., Lindmark B. (2019). Accelerated drug discovery by rapid candidate drug identification. Drug Discov. Today.

[48] Lee W.H. (2015). Open access target validation is a more efficient way to accelerate drug discovery. PLoS Biol..

[49] Yao R., Shen Z., Xu X., Ling G., Xiang R., Song T., Zhai F., Zhai Y. (2024). Knowledge mapping of graph neural networks for drug discovery: A bibliometric and visualized analysis. Front. Pharmacol..

[50] Lin X., Quan Z., Wang Z.J., Ma T., Zeng X. (2020). KGNN: Knowledge graph neural network for drug-drug interaction prediction. IJCAI’20: Proceedings of the Twenty-Ninth International Conference on International Joint Conferences on Artificial Intelligence.

[51] Su C., Hou Y., Wang F. (2022). GNN-based Biomedical Knowledge Graph mining in drug development. Graph Neural Networks: Foundations, Frontiers, and Applications.

[52] Zhang O., Lin H., Zhang X., Wang X., Wu Z., Ye Q., Zhao W., Wang J., Ying K., Kang Y. (2025). Graph neural networks in modern AI-aided drug discovery. Chem. Rev..

[53] Chen S., Semenov I., Zhang F., Yang Y., Geng J., Feng X., Meng Q., Lei K. (2024). An effective framework for predicting drug–drug interactions based on molecular substructures and knowledge graph neural network. Comput. Biol. Med..

[54] Martis E.A., Radhakrishnan R., Badve R.R. (2011). High-throughput screening: The hits and leads of drug discovery-an overview. J. Appl. Pharm. Sci..

[55] Szymański P., Markowicz M., Mikiciuk-Olasik E. (2011). Adaptation of high-throughput screening in drug discovery—Toxicological screening tests. Int. J. Mol. Sci..

[56] Hajare A.A., Salunkhe S.S., Mali S.S., Gorde S.S., Nadaf S.J., Pishawikar S.A. (2013). Review on: High-throughput screening is an approach to drug discovery. Am. J. Pharm. Tech. Res..

[57] Carnero A. (2006). High throughput screening in drug discovery. Clin. Transl. Oncol..

[58] Aldewachi H., Al-Zidan R.N., Conner M.T., Salman M.M. (2021). High-throughput screening platforms in the discovery of novel drugs for neurodegenerative diseases. Bioengineering.

[59] Roy A. (2022). High-Throughput screening (HTS) technology. Encyclopedia of Molecular Pharmacology.

[60] Barcelos M.P., Gomes S.Q., Federico L.B., Francischini I.A.G., Hage-Melim L.I.D.S., Silva G.M., de Paula da Silva C.H.T. (2022). Lead optimization in drug discovery. Research Topics in Bioactivity, Environment and Energy: Experimental and Theoretical Tools.

[61] Showell G.A., Mills J.S. (2003). Chemistry challenges in lead optimization: Silicon isosteres in drug discovery. Drug Discov. Today.

[62] Heifetz A., Southey M., Morao I., Townsend-Nicholson A., Bodkin M.J. (2017). Computational methods used in hit-to-lead and lead optimization stages of structure-based drug discovery. Computational Methods for GPCR Drug Discovery.

[63] Zhang J., Li H., Zhang Y., Huang J., Ren L., Zhang C., Zou Q., Zhang Y. (2025). Computational toxicology in drug discovery: Applications of artificial intelligence in ADMET and toxicity prediction. Brief. Bioinform..

[64] Swanson K., Walther P., Leitz J., Mukherjee S., Wu J.C., Shivnaraine R.V., Zou J. (2024). ADMET-AI: A machine learning ADMET platform for evaluation of large-scale chemical libraries. Bioinformatics.

[65] Biehn S.E., Goncalves L.M., Lehmann J., Marty J.D., Mueller C., Ramirez S.A., Tillier F., Sage C.R. (2024). BioPrint meets the AI age: Development of artificial intelligence-based ADMET models for the drug-discovery platform SAFIRE. Future Med. Chem..

[66] Han R., Yoon H., Kim G., Lee H., Lee Y. (2023). Revolutionizing medicinal chemistry: The application of artificial intelligence (AI) in early drug discovery. Pharmaceuticals.

[67] Staszak M., Staszak K., Wieszczycka K., Bajek A., Roszkowski K., Tylkowski B. (2022). Machine learning in drug design: Use of artificial intelligence to explore the chemical structure–biological activity relationship. Wiley Interdiscip. Rev. Comput. Mol. Sci..

[68] Bhisetti G., Fang C. (2021). Artificial intelligence–enabled de novo design of novel compounds that are synthesizable. Artificial Intelligence in Drug Design.

[69] Walters W.P., Barzilay R. (2021). Critical assessment of AI in drug discovery. Expert Opin. Drug Discov..

[70] Rohith Reddy G.K., Ibrahim S.A., Ali S.J. (2026). ADMET-X: Machine Learning Framework for Predicting Pharmacokinetic and Toxicological Drug Properties. 2026 International Conference on AI-Driven Smart Systems and Ubiquitous Computing (ICAUC).

[71] Tanihata S., Iwata H. (2026). Next-Generation Artificial Intelligence for ADME Prediction in Drug Discovery: From Small Molecules to Biologics. Yonago Acta Medica.

[72] Ali S., Tian X., Chen H., Zhou J. (2025). A New Era of Artificial Intelligence (AI): Transforming Drug Discovery and Development. J. Med. Chem..

[73] Swanson K., Mukherjee S., Walther P., Lai C., Yan C., Shivnaraine R., Leitz J., Pang P., Zou J., Wu J. (2024). ADMET-AI enables interpretable predictions of drug-induced cardiotoxicity. Circulation.

[74] Bender A., Cortés-Ciriano I. (2021). Artificial intelligence in drug discovery: What is realistic, what are illusions? Part 1: Ways to make an impact, and why we are not there yet. Drug Discov. Today.

[75] Mak K.K., Wong Y.H., Pichika M.R. (2024). Artificial intelligence in drug discovery and development. Drug Discovery and Evaluation: Safety and Pharmacokinetic Assays.

[76] Michael S., Auld D., Klumpp C., Jadhav A., Zheng W., Thorne N., Austin C.P., Inglese J., Simeonov A. (2008). A robotic platform for quantitative high-throughput screening. Assay. Drug Dev. Technol..

[77] Zhu M. (2015). A Review on Recent Robotic and Analytic Technologies in High Throughput Screening and Synthesis for Drug Discovery. Lett. Drug Des. Discov..

[78] Nelsen L.L. (2005). The role of research institutions in the formation of the biotech cluster in Massachusetts: The MIT experience. J. Commer. Biotechnol..

[79] Sarkar C., Das B., Rawat V.S., Wahlang J.B., Nongpiur A., Tiewsoh I., Lyngdoh N.M., Das D., Bidarolli M., Sony H.T. (2023). Artificial intelligence and machine learning technology driven modern drug discovery and development. Int. J. Mol. Sci..

[80] Tiwari G., Malik A., Singh B., Shukla K., Sachan P., Tiwari R., Gupta A.K., Hema G., Singh G.H.A. (2022). Artificial Intelligence and Robotics: A Step Forward Towards Drug Discovery. Neuroquantology.

[81] Vamathevan J., Clark D., Czodrowski P., Dunham I., Ferran E., Lee G., Li B., Madabhushi A., Shah P., Spitzer M. (2019). Applications of machine learning in drug discovery and development. Nat. Rev. Drug Discov..

[82] Li D., Hu J., Zhang L., Li L., Yin Q., Shi J., Zhuang P. (2022). Deep learning and machine intelligence: New computational modeling techniques for discovery of the combination rules and pharmacodynamic characteristics of Traditional Chinese Medicine. Eur. J. Pharmacol..

[83] Khan S.R., Al Rijjal D., Piro A., Wheeler M.B. (2021). Integration of AI and traditional medicine in drug discovery. Drug Discov. Today.

[84] Farea A., Yli-Harja O., Emmert-Streib F. (2024). Understanding physics-informed neural networks: Techniques, applications, trends, and challenges. AI.

[85] Huang B., Wang J. (2022). Applications of physics-informed neural networks in power systems-a review. IEEE Trans. Power Syst..

[86] Zhang M., Wu H., Wang Y. (2024). Enhanced sampling of biomolecular slow conformational transitions using adaptive sampling and machine learning. J. Chem. Theory Comput..

[87] Jiménez-Boi R., Miñán R., Pallara C., Lozoya E., Guallar V., Molina A., Soliva R. (2025). How Feasible Is Docking of PROTACs to POI-E3L Complexes? Testing Physics-Based and ML-Based Docking Tools. J. Chem. Inf. Model..

[88] Parvatikar P.P., Patil S., Khaparkhuntikar K., Patil S., Singh P.K., Sahana R., Kulkarni R.V., Raghu A.V. (2023). Artificial intelligence: Machine learning approach for screening large database and drug discovery. Antivir. Res..

[89] Taha M.O., Daoud S. (2026). Innovative integration of molecular docking and machine learning for drug discovery: From virtual screening to nanomolar inhibitors. Chem. Commun..

[90] Thaingtamtanha T., Ravichandran R., Gentile F. (2025). On the application of artificial intelligence in virtual screening. Expert Opin. Drug Discov..

[91] Nasim I., Nasim A. (2024). Discovering intrinsic multi-compartment pharmacometric models using Physics Informed Neural Networks. arXiv.

[92] Awojoyogbe B.O., Dada M.O. (2024). Physics Informed Neural Networks (PINNs). Digital Molecular Magnetic Resonance Imaging.

[93] Azam F., Almahmoud S.A. (2026). Open-Source Molecular Docking and AI-Augmented Structure-Based Drug Design: Current Workflows, Challenges, and Opportunities. Int. J. Mol. Sci..

[94] Khurshid B., Khurshid S., Rafique A.M., Javaid M. (2025). AI for Drug Discovery: From Algorithms to Medicines. J. Pharm. Biomed..

[95] Renaud J.P., Chung C.W., Danielson U.H., Egner U., Hennig M., Hubbard R.E., Nar H. (2016). Biophysics in drug discovery: Impact, challenges and opportunities. Nat. Rev. Drug Discov..

[96] Kakraba S., Agyemang E.F., Reis R.J.S. (2026). Accelerating Discovery of Leukemia Inhibitors Using AI-Driven Quantitative Structure-Activity Relationship: Algorithm Development and Validation. JMIR AI.

[97] Pitt W.R., Bentley J., Boldron C., Colliandre L., Esposito C., Frush E.H., Kopec J., Labouille S., Meneyrol J., Pardoe D.A. (2025). Real-World Applications and Experiences of AI/ML Deployment for Drug Discovery. J. Med. Chem..

[98] Jiao R., Zhang S., Zhang H., Deng B., Zhang T., Tang S., Hu X., Zhang W. (2026). Physics–Data-Integrated Hybrid Simulation for Transient Stability in New Power Systems: Status, Challenges, and Prospects. Energies.

[99] Kotsis K. (2025). Integrating Artificial Intelligence into the Higher Education of Physics: Theoretical Frameworks and Pedagogical Strategies. Gabaldon Int. J. Educ. Methodol..

[100] Grassano J.S., Pickering I., Roitberg A.E., Estrin D.A., Semelak J.A. (2025). From QM/MM to ML/MM: A new era in multiscale modeling. Chem. Phys. Rev..

[101] Cui T., Zhou Y., Wang T. (2025). Recent advances in artificial intelligence–driven biomolecular dynamics simulations based on machine learning force fields. Curr. Opin. Struct. Biol..

[102] Wang Z., Wu H., Sun L., He X., Liu Z., Shao B., Wang T., Liu T.-Y. (2023). Improving machine learning force fields for molecular dynamics simulations with fine-grained force metrics. J. Chem. Phys..

[103] Huang K., Fu T., Gao W., Zhao Y., Roohani Y., Leskovec J., Coley C.W., Xiao C., Sun J., Zitnik M. (2021). Therapeutics data commons: Machine learning datasets and tasks for drug discovery and development. arXiv.

[104] Niazi S.K. (2025). Quantum mechanics in drug discovery: A comprehensive review of methods, applications, and future directions. Int. J. Mol. Sci..

[105] Rowaiye A.B., Folarin A.A., Akingbade T., Okoli J.C., Rowaiye O.I., Folorunso T.R., Bur D. (2025). Advancing predictive modeling in computational chemistry through quantum chemistry, molecular mechanics, and machine learning. Discov. Chem..

[106] Catlow C.R.A., Buckeridge J., Farrow M.R., Logsdail A.J., Sokol A.A. (2017). Quantum Mechanical/Molecular Mechanical (QM/MM) Approaches.

[107] Rothlisberger U., Carloni P. (2006). Drug-target binding investigated by quantum mechanical/molecular mechanical (QM/MM) methods. Computer Simulations in Condensed Matter Systems: From Materials to Chemical Biology Volume 2.

[108] Al-Dahlaki M.H., Mohammed A.H. (2024). Quantum mechanics/molecular mechanics (QM/MM) methods in drug design: A comprehensive review of development and applications. Int. J. Adv. Chem..

[109] Barbault F., Maurel F. (2015). Simulation with quantum mechanics/molecular mechanics for drug discovery. Exper. Opin. Drug Discov..

[110] Van Der Kamp M.W., Mulholland A.J. (2013). Combined quantum mechanics/molecular mechanics (QM/MM) methods in computational enzymology. Biochemistry.

[111] Kim H., Kim E., Lee I., Bae B., Park M., Nam H. (2020). Artificial intelligence in drug discovery: A comprehensive review of data-driven and machine learning approaches. Biotechnol. Bioprocess. Eng..

[112] Ash J.R., Wognum C., Rodríguez-Pérez R., Aldeghi M., Cheng A.C., Clevert D.A., Walters W.P. (2025). Practically significant method comparison protocols for machine learning in small molecule drug discovery. J. Chem. Inf. Model..

[113] Udegbe F.C., Ebulue O.R., Ebulue C.C., Ekesiobi C.S. (2024). Machine Learning in Drug Discovery: A critical review of applications and challenges. Comput. Sci. IT Res. J..

[114] Zhang L., Tan J., Han D., Zhu H. (2017). From machine learning to deep learning: Progress in machine intelligence for rational drug discovery. Drug Discov. Today.

[115] Qi X., Zhao Y., Qi Z., Hou S., Chen J. (2024). Machine learning empowering drug discovery: Applications, opportunities and challenges. Molecules.

[116] Kumar S.A., Ananda Kumar T.D., Beeraka N.M., Pujar G.V., Singh M., NarayanaAkshatha H.S., Bhagyalalitha M. (2022). Machine learning and deep learning in data-driven decision making of drug discovery and challenges in high-quality data acquisition in the pharmaceutical industry. Future Med. Chem..

[117] Dara S., Dhamercherla S., Jadav S.S., Babu C.M., Ahsan M.J. (2022). Machine learning in drug discovery: A review. Artif. Intell. Rev..

[118] Singh S., Kumar R., Payra S., Singh S.K. (2023). Artificial intelligence and machine learning in pharmacological research: Bridging the gap between data and drug discovery. Cureus.

[119] Peña-Guerrero J., Nguewa P.A., García-Sosa A.T. (2021). Machine learning, artificial intelligence, and data science breaking into drug design and neglected diseases. Wiley Interdiscip. Rev. Comput. Mol. Sci..

[120] Nayarisseri A., Khandelwal R., Tanwar P., Madhavi M., Sharma D., Thakur G., Singh S.K. (2021). Artificial intelligence, big data and machine learning approaches in precision medicine & drug discovery. Curr. Drug Targets.

[121] Ponce-Bobadilla A.V., Schmitt V., Maier C.S., Mensing S., Stodtmann S. (2024). Practical guide to SHAP analysis: Explaining supervised machine learning model predictions in drug development. Clin. Transl. Sci..

[122] Miao K., Hounye A.H., Su L., Pan Q., Wang J., Hou M., Xiong L. (2024). Exploring explainable machine learning and Shapley additive exPlanations (SHAP) technique to uncover key factors of HNSC cancer: An analysis of the best practices. Biomed. Signal Process. Control.

[123] Sanjaya A., Ratnawati H., Mianto N.A., Camillo K.V., Tedjo A., Kusuma W.A. (2025). Enhancing Quantitative Structure--Activity Relationship Predictive Power and Explainability: Meta-Modeling and Shapley Additive Explanations Feature Importance Analysis for Drug Discovery. Trop. J. Nat. Prod. Res..

[124] Lavecchia A. (2019). Deep learning in drug discovery: Opportunities, challenges and future prospects. Drug Discov. Today.

[125] Shan W., Xu J., Assaraf Y.G. (2026). Data-driven targetome discovery and database requirements: Insights from the therapeutic target database. Targetome.

[126] Mallon A.-M., Häring D.A., Dahlke F., Aarden P., Afyouni S., Delbarre D., El Emam K., Ganjgahi H., Gardiner S., Kwok C.H. (2021). Advancing data science in drug development through an innovative computational framework for data sharing and statistical analysis. BMC Med. Res. Methodol..

[127] Weise A., Buechter R., Pieper D., Mathes T. (2020). Assessing context suitability (generalizability, external validity, applicability or transferability) of findings in evidence syntheses in healthcare—An integrative review of methodological guidance. Res. Synth. Methods.

[128] Huang D.Z., Baber J.C., Bahmanyar S.S. (2021). The challenges of generalizability in artificial intelligence for ADME/Tox endpoint and activity prediction. Expert Opin. Drug Discov..

[129] Xia X., Zhu C., Zhong F., Liu L. (2024). TransCDR: A deep learning model for enhancing the generalizability of drug activity prediction through transfer learning and multimodal data fusion. BMC Biol..

[130] Thakkar S., SlikkerJr W., Yiannas F., Silva P., Blais B., Chng K.R., Tong W. (2023). Artificial intelligence and real-world data for drug and food safety–A regulatory science perspective. Regul. Toxicol. Pharmacol..

[131] Lenarczyk G., Minssen T., Price N., Rai A. (2025). The future of AI regulation in drug development: A comparative analysis. J. Law Biosci..

[132] Teixeira T., Kweder S.L., Saint-Raymond A. (2020). Are the European Medicines Agency, US Food and Drug Administration, and other international regulators talking to each other?. Clin. Pharmacol. Ther..

[133] Singh V., Cheng S., Kwan A.C., Ebinger J. (2025). United States Food and Drug Administration regulation of clinical software in the era of artificial intelligence and machine learning. Mayo Clin. Proc. Digit. Health.

[134] Hu Z., Bhutto J.A., Ishfaq M., Shah S.W.A., Bilawal A., Shah Z., Guan Y. (2025). Ethical considerations in the application of machine learning and artificial intelligence in medicinal chemistry, pharmacology, and toxicology. Computational Methods in Medicinal Chemistry, Pharmacology, and Toxicology.

[135] Anastasopoulou C., Efthymiou I.P. (2024). Ethical Issues Arising from the Use of AI in Drug Discovery. J. Politics Ethics New Technol. Artif. Intell..

[136] Al-Worafi Y.M. (2023). Artificial intelligence and machine learning for drug safety. Technology for Drug Safety: Current Status and Future Developments.

[137] Arabi A.A. (2021). Artificial intelligence in drug design: Algorithms, applications, challenges and ethics. Future Drug Discov..

[138] Watson J., AHutyra C., Clancy S.M., Chandiramani A., Bedoya A., Ilangovan K., Nderitu N., Poon E.G. (2020). Overcoming barriers to the adoption and implementation of predictive modeling and machine learning in clinical care: What can we learn from US academic medical centers?. JAMIA Open.

[139] Chakraborty C., Bhattacharya M., Lee S.S., Wen Z.H., Lo Y.H. (2024). The changing scenario of drug discovery using AI to deep learning: Recent advancement, success stories, collaborations, and challenges. Mol. Ther. Nucleic Acids.

[140] Ferreira F.J., Carneiro A.S. (2025). AI-driven drug discovery: A comprehensive review. ACS Omega.

[141] Pache M.M., Pangavhane R.R., Jagtap M.N., Darekar A.B. (2025). The AI-driven future of drug discovery: Innovations, applications, and challenges. Asian J. Res. Pharm. Sci..

[142] Ozaybi M.Q.B., Madkhali A.N.M., Alhazmi M.A.M., Faqihi H.M.A., Alanazi M.M., Siraj W.H.Y., Zalah A.H.A., Khormi M.M.A., al Salem A.M.A., Mashragi T.Q.M. (2024). The role of artificial intelligence in drug discovery and development. Egypt. J. Chem..

[143] Patel R., Patel A. (2024). Revolutionizing drug development: AI-driven predictive modeling for accelerated small molecule and biologic therapeutics. Well Test. J..

[144] Koirala M., Yan L., Mohamed Z., DiPaola M. (2025). AI-Integrated QSAR modeling for enhanced drug discovery: From classical approaches to deep learning and structural insight. Int. J. Mol. Sci..

[145] Enni M.A., Maraj M.A.A. (2022). In Silico drug repurposing for inflammatory diseases: A systematic review of molecular docking and virtual screening studies. Am. J. Adv. Technol. Eng. Solut..

[146] Gangwal A., Ansari A., Ahmad I., Azad A.K., Kumarasamy V., Subramaniyan V., Wong L.S. (2024). Generative artificial intelligence in drug discovery: Basic framework, recent advances, challenges, and opportunities. Front. Pharmacol..

[147] Lavecchia A. (2025). Transform drug discovery and development with generative artificial intelligence. Generative Artificial Intelligence for Biomedical and Smart Health Informatics.

[148] Manik M.M.T.G., Bhuiyan M.M.R., Moniruzzaman M., Islam M.S., Hossain S., Hossain S. (2018). The future of drug discovery utilizing generative AI and big data analytics for accelerating pharmaceutical innovations. Nanotechnol. Percept..

[149] Das U. (2025). Transforming precision medicine through generative AI: Advanced architectures and tailored therapeutic design for patient-specific drug discovery. ChemistrySelect.

[150] Terranova N., Venkatakrishnan K., Benincosa L.J. (2021). Application of machine learning in translational medicine: Current status and future opportunities. AAPS J..

[151] Sharma D., Anabala M., Jain V.V., Shyam M., Prince S.E., Muniyan R. (2025). Computational Landscape in Drug Discovery: From AI/ML Models to Translational Application. Scientifica.

[152] Naik K., Goyal R.K., Foschini L., Chak C.W., Thielscher C., Zhu H., Lu J., Lehár J., Pacanoswki M.A., Terranova N. (2024). Current status and future directions: The application of artificial intelligence/machine learning for precision medicine. Clin. Pharmacol. Ther..

[153] Uddin M.R., Sovon M.S.I., Mondal S., Ahmed S., Al-Mizan M.A., Aktar F., Amran M.S. (2025). Artificial intelligence and machine learning in pharmaceutical sciences: Unpacking regulatory guidance, opportunities, and challenges for safe and effective drug development. Int. J. Med. Inform..

[154] Agarwal B., Gaware S., More N., Shinde R., Shivakumar H.N., Jagdale S. (2026). A Review Exploring the Translational Perspective of Artificial Intelligence in Drug Discovery and Formulation Development. Ann. Pharm. Françaises.

[155] Bhattacharya M., Pal S., Chatterjee S., Lee S.S., Chakraborty C. (2024). Large language model to multimodal large language model: A journey to shape the biological macromolecules to biological sciences and medicine. Mol. Ther. Nucleic Acids.

[156] Yan L., Jiang X., Ma J., Liu Y., Bian T., Wang Q., Basu A., Rong Y., Xu T., Wu P. A Comprehensive Survey of Multimodal LLMs for Scientific Discovery. Proceedings of the 1st Workshop on VLM4RWD@ NeurIPS 2025.

[157] Buess L., Keicher M., Navab N., Maier A., TayebiArasteh S. (2025). From large language models to multimodal AI: A scoping review on the potential of generative AI in medicine. Biomed. Eng. Lett..

[158] Ma J., Liu J., Xu D., Song X., Zhang Z. (2025). Application and Prospects of Large Language Models in Small-Molecule Drug Discovery. Anal. Chem..

[159] Ye G. (2024). De novo drug design as GPT language modeling: Large chemistry models with supervised and reinforcement learning. J. Comput.-Aided Mol. Des..

[160] Gangwal A., Lavecchia A. (2026). Large Language Models in Drug Discovery. Applied Artificial Intelligence for Drug Discovery: From Data-Driven Insights to Therapeutic Innovation.

[161] Rodrigues T., Bernardes G.J. (2020). Machine learning for target discovery in drug development. Curr. Opin. Chem. Biol..

[162] He J., Hua C., Wang Y., Zheng Z. (2025). Collaborative intelligence in sequential experiments: A human-in-the-loop framework for drug discovery. Inf. Syst. Res..

[163] Shi C., Ren X., Wang Y., Li J., Sun Y., Luo Y., Sheng R. (2026). A Survey of Human-AI Collaboration for Scientific Discovery. Preprints.org.

[164] Wang D., Churchill E., Maes P., Fan X., Shneiderman B., Shi Y., Wang Q. (2020). From human-human collaboration to Human-AI collaboration: Designing AI systems that can work together with people. Extended Abstracts of the 2020 CHI Conference on Human Factors in Computing Systems.

[165] Wang L., Zhou Z., Yang X., Shi S., Zeng X., Cao D. (2024). The present state and challenges of active learning in drug discovery. Drug Discov. Today.

[166] Avramouli M., Savvas I.K., Vasilaki A., Garani G. (2023). Unlocking the potential of quantum machine learning to advance drug discovery. Electronics.

[167] Arvidsson P.I., Sandberg K., Forsberg-Nilsson K. (2016). Open for collaboration: An academic platform for drug discovery and development at SciLifeLab. Drug Discov. Today.

[168] Saini J.P.S., Thakur A., Yadav D. (2025). AI-driven innovations in pharmaceuticals: Optimizing drug discovery and industry operations. RSC Pharm..

[169] Blanco-Gonzalez A., Cabezon A., Seco-Gonzalez A., Conde-Torres D., Antelo-Riveiro P., Pineiro A., Garcia-Fandino R. (2023). The role of AI in drug discovery: Challenges, opportunities, and strategies. Pharmaceuticals.

[170] Garg S., Arora K., Singh S., Nagarajan K. (2024). Artificial intelligence and machine learning in drug discovery and development. Artificial Intelligence in the Age of Nanotechnology.

[171] Visan A.I., Negut I. (2024). Integrating artificial intelligence for drug discovery in the context of revolutionizing drug delivery. Life.

[172] Vora L.K., Gholap A.D., Jetha K., Thakur R.R.S., Solanki H.K., Chavda V.P. (2023). Artificial intelligence in pharmaceutical technology and drug delivery design. Pharmaceutics.

[173] Aundhia C., Parmar G., Talele C., Shah N., Talele D. (2025). Impact of artificial intelligence on drug development and delivery. Curr. Top. Med. Chem..

[174] Asediya V., Anjaria P., Dhial K., Pathak A. (2025). Artificial Intelligence and Machine Learning in Drug Delivery Optimization. Next-Generation Drug Delivery Systems.

[175] Rabbani S.A., El-Tanani M., Sharma S., Rabbani S.S., El-Tanani Y., Kumar R., Saini M. (2025). Generative artificial intelligence in healthcare: Applications, implementation challenges, and future directions. BioMedInformatics.

[176] Rabbani S.A., El-Tanani M., El-Tanani Y., Kumar R., Sharma S., Khan M.A., Parvez S., Aljabali A.A.A., Matalka M.I., Rizzo M. (2025). Advances in Adoptive Cell Therapies in Cancer: From Mechanistic Breakthroughs to Clinical Frontiers and Overcoming Barriers. Med. Sci..

